# Single-Source Alkoxide Precursor Approach to Titanium Molybdate, TiMoO_5_, and Its Structure, Electrochemical Properties, and Potential as an Anode Material for Alkali Metal Ion Batteries

**DOI:** 10.1021/acs.inorgchem.0c03087

**Published:** 2021-02-22

**Authors:** Hiroaki Uchiyama, Dhanya Puthusseri, Jekabs Grins, Daniel Gribble, Gulaim A. Seisenbaeva, Vilas G. Pol, Vadim G. Kessler

**Affiliations:** † Department of Molecular Sciences, BioCenter, Swedish University of Agricultural Sciences, Box 7015, SE-750 07 Uppsala, Sweden; ‡ Kansai University, 3-3-35 Yamate-cho, Suita-shi, Osaka 564-8680, Japan; § Davidson School of Chemical Engineering, Purdue University, West Lafayette, Indiana 47907, United States; ∥ Department of Materials and Environmental Chemistry, Stockholm University, SE-106 91 Stockholm, Sweden

## Abstract

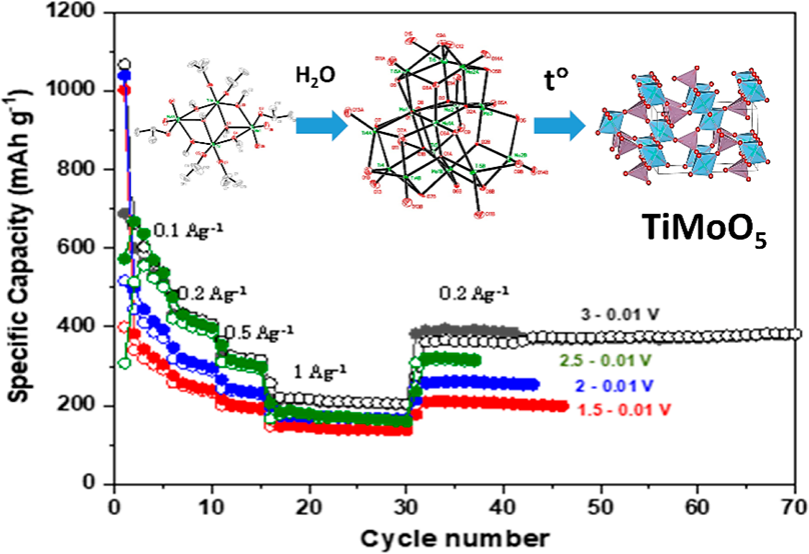

Transition-metal oxide nanostructured materials are potentially attractive alternatives as anodes for Li ion batteries and as photocatalysts. Combining the structural and thermal stability of titanium oxides with the relatively high oxidation potential and charge capacity of molybdenum(VI) oxides was the motivation for a search for approaches to mixed oxides of these two metals. Challenges in traditional synthetic methods for such materials made development of a soft chemistry single-source precursor pathway our priority. A series of bimetallic Ti-Mo alkoxides were produced by reactions of homometallic species in a 1:1 ratio. Thermal solution reduction with subsequent reoxidation by dry air offered in minor yields Ti_2_Mo_2_O_4_(OMe)_6_(O^i^Pr)_6_ (**1**) by the interaction of Ti(O^i^Pr)_4_ with MoO(OMe)_4_ and Ti_6_Mo_6_O_22_(O^i^Pr)_16_(iPrOH)_2_ (**2**) by the reaction of Ti(O^i^Pr)_4_ with MoO(O^i^Pr)_4_. An attempt to improve the yield of **2** by microhydrolysis, using the addition of stoichiometric amounts of water, resulted in the formation with high yield of a different complex, Mo_7_Ti_7+*x*_O_31+*x*_(O^i^Pr)_8+2*x*_ (**3**). Controlled thermal decomposition of **1**–**3** in air resulted in their transformation into the phase TiMoO_5_ (**4**) with an orthorhombic structure in space group *Pnma*, as determined by a Rietveld refinement. The electrochemical characteristics of **4** and its chemical transformation on Li insertion were investigated, showing its potential as a promising anode material for Li ion batteries for the first time. A lower charge capacity and lower stability were observed for its application as an anode for a Na ion battery.

## Abstract 2

Controlled decomposition of a structurally characterized Ti-Mo oxo-alkoxide precursor offered an approach to the new ternary oxide TiMoO_5_, showing promising characteristics as an anode material for Li ion batteries.

## Introduction

The interest in early-transition-metal oxide materials has in recent years been fueled by perspectives of their application in photovoltaics,^1^ as photocatalysts in the production of solar fuels,^2^ and as electrodes for alkali-metal batteries.^3^ A special place among the target compounds belongs to titanium dioxide and titanates. The photocatalytic properties of TiO_2_ were discovered by Honda and Fujishima,^4^ who pioneered this field. Titania was shown also to be prospective as a robust anode material for Li ion batteries, competing with graphite due to its reasonable charge capacity (ca. 200 mAh/g) and ability to prevent the formation of lithium dendrites.^5,6^ Nanostructured molybdenum trioxide has also been proved to possess excellent photocatalytic properties when it is irradiated by visible light.^7^ MoO_3_ was actually proved to possess an outstanding charge capacity as a Li ion battery anode (over 1000 mAh/g) but displayed rather high chemical reactivity, leading to an easy change in morphology, challenging its application for this purpose.^8^

It appeared therefore very attractive to investigate the functional characteristics of a mixed-metal titanium–molybdenum oxide. In the literature, the information about such materials is very scarce and not fully reliable. The solubility of TiO_2_ and MoO_3_ in each other is very limited.^9^ The only mixed-metal oxide compound, TiMoO_5_, has been reported in patent literature.^10^ It was proposed to possess a tetragonal structure in the ICDD X-ray powder database,^11^ but no crystal structure determination was reported. The synthesis of a mixed titanium–molybdenum oxide by traditional solid-state or solution techniques is quite challenging in view of the rather different thermal stabilities, acidities, and solubilities of TiO_2_ and MoO_3_. An attractive alternative to traditional synthetic approaches in this case seemed the single-source precursor route, exploiting an easily decomposable mixed-metal precursor. This approach can be effective only if the target oxide phase is stable under the given conditions,^12^ but the few reports on the existence of the compound TiMoO_5_ gave us hope for success.

Mixed-metal alkoxides are often seen as suitable precursors, undergoing thermal decomposition to oxides under extremely mild conditions, most often in the range 150–180 °C.^13^ Even the reports on bimetal titanium–molybdenum alkoxide derivatives appeared very scarce in the literature, with only two compounds, (^n^Bu_4_N)_3_[(^i^PrO)TiMo_5_O_18_]^14,15^ and [{Ti_4_Mo^V^_2_O_8_(OEt)_10_}_2_],^16^ having compositions not corresponding to that of the target oxide phase. We saw therefore as our aim to develop synthetic approaches and produce structural characterization for bimetallic titanium–molybdenum alkoxides with a 1:1 composition, utilize them in the synthesis of a TiMoO_5_ material, and finally, investigate its structure and electrochemical properties to verify its potential applicability as an anode material for Li ion batteries.

## Experimental Section

All operations with alkoxide precursor compounds were carried out in a dry nitrogen atmosphere using a Schlenk line or drybox. The starting reagents of p.a. grade, Ti(O^i^Pr)_4_, Mo metal, methanol and 2-propanol, as well as toluene applied as solvent, were purchased from Sigma-Aldrich Sweden AB. Methanol was purified by distillation over Mg(OMe)_2_, synthesized *in situ* by interaction of Mg turnings with MeOH on addition of minor amounts of solid iodine as a catalyst, while 2-propanol was distilled over Al(O^i^Pr)_3_ obtained by an analogous procedure. Toluene was dried by distillation over LiAlH_4_. MoO(OMe)_4_ was produced by anodic oxidation of Mo metal in methanol following a procedure described earlier,^17^ and MoO(O^i^Pr)_4_ was prepared using an alcohol interchange technique as previously reported.^18^

### Ti_2_Mo_2_O_4_(OMe)_6_(O^i^Pr)_6_ (**1**)

Ti(O^i^Pr)_4_ (102 mg, 0.357 mmol) and MoO(OMe)_4_ (133 mg, 0.563 mmol) were added to 1.0 mL of toluene and then refluxed for 20 min. The brownish solution obtained was left in a freezer (−18 °C) for several days, producing a very small crop of thin needle-shaped crystals. The reaction mixture was warmed until complete dissolution of the initial crystals, and then left for oxidation and concentration connected to air via a column filled with dry 4 Å molecular sieves. A few block-shaped crystals formed in the residual syruplike liquid. Their composition was identified by an X-ray single-crystal study as Ti_2_Mo_2_O_4_(OMe)_6_(O^i^Pr)_6_ (**1**).

### Ti_6_Mo_6_O_22_(O^i^Pr)_16_(iPrOH)_2_ (**2**)

Ti(O^i^Pr)_4_ (385 mg, 1.35 mmol) and MoO(O^i^Pr)_4_ (446 mg, 1.28 mmol) was dissolved in 0.5 mL toluene and then the solution was refluxed for 15 min. The brown solution that was produced was left in a freezer (−18 °C) overnight, and then a brown amorphous product precipitated. A 3 mL portion of hexane was added onto the brown amorphous material, resulting in a brown liquid and an amorphous brownish powder. The latter was separated by decantation, and then the residual brown liquid was kept in a polypropylene syringe at room temperature for several days, producing colorless crystals. This product was identified as Ti_6_Mo_6_O_22_(O^i^Pr)_16_(^i^PrOH)_2_ (**2**) by an X-ray single-crystal study.

### Mo_7_Ti_7+*x*_O_31+*x*_(O^i^Pr)_8+2*x*_ (**3**)

Ti(O^i^Pr)_4_ (2.08 g, 7.32 mmol) and MoO(O^i^Pr)_4_ (2.60 g, 7.45 mmol) were added to 10 mL of toluene, resulting in a yellow solution. A mixture of H_2_O (0.35 g, 19.4 mmol) and 10 mL of 2-propanol was added to the yellow solution, and then the resulting solution was left at room temperature for several days, the solution color changing then to blue and colorless single crystals precipitating (ca. 1.07 g). The product was identified as Mo_7_Ti_7+*x*_O_31+*x*_(O^i^Pr)_8+2*x*_ (**3**) by an X-ray single crystal study. The metal ratio in the crystals was Ti:Mo = 1:1 according to SEM-EDS analysis. The single crystals were removed by decantation, and then the solvent was evaporated from the decanted solution to half its volume. The resulting liquid was left in a freezer (−18 °C) for several days and then at room temperature for several days, leading to the precipitation of an additional amount of the same crystalline product (0.719 g), as proved by a unit cell determination of several single crystals and microscopic (optical and SEM) observations. The total yield was ca. 86%. FTIR, cm^–1^: 1711 s, 1162 w, 1131 w, 1023 sh, 956 m, 934 m, 914 m, 773 w, 721 m, 677 w, 595 m br. ^1^H NMR, δ, ppm: septets 5.05 1H, 4.95 1H, 4.52 1H, 4.02 about 4H, C*H*-^i^Pr; 1.47 6H, 1.42 6H, 1.33 6H, 1.30 9H, 1.20 12H, 1.15 3H, C*H*_3_-^i^Pr. ^13^C NMR, δ, ppm: 81.5, 79.5, 75.5, 64.5, 62.5, *C*H-^i^Pr; 25.72, 25.45, 25.38, 24.53, 23.48, 22.82, 20.54, 20.10, *C*H_3_-^i^Pr.

### TiMoO_5_ (**4**)

Colorless crystals of **3** obtained previously (ca. 0.87 g) were heated from 25 to 400 °C at 10 °C/min and then up to 500 °C at 3 °C/min, followed by holding at 500 °C for 30 min. After the heating, a bluish coarse powder was obtained. The bluish powder was milled with a mortar and then heated again from 25 to 400 °C at 10 °C/min and then up to 500 °C at 3 °C/min, followed by holding at 500 °C for 2 h. After the second heating, a yellowish powder of **4** was obtained (ca. 0.46 g, ceramic yield 53%). The metal ratio in the yellowish powder was Ti:Mo = 1:1 according to XPS and SEM-EDS analyses. The nature of **4** was confirmed by a structure determination using Rietveld refinement for X-ray powder diffraction data.

### Characterization

IR spectra of Nujol mulls were registered with a PerkinElmer Spectrum 100 FT-IR spectrometer. ^1^H and ^13^C NMR spectra were obtained for solutions in anhydrous CDCl_3_ with a Bruker 600 MHz spectrometer.

An SEM-EDS study was performed using a Hitachi TM-1000-μ-DeX tabletop electron microscope and Flex-SEM 1000 scanning electron microscope. Transmission electron microscopy (TEM) images were taken with a JEOL JEM-2100F Schottky field-emission microscope operated at 200 kV (Cs = 0.5 mm) and equipped with a Gatan Ultrascan 1000 CCD camera, a postcolumn imaging filter (Gatan Tridiem 863), and a Gatan annular dark-field (ADF) detector. The edge position of each element (Si L-edge, O K-edge, and the M4,5-edge of La, Nd ,and Dy) was determined by electron energy loss spectroscopy (EELS) in advance. The results of elemental mapping and thickness variation are presented in pseudocolor. The metal ratio was measured using an X-ray photoelectron spectrometer (PHI5000 Versa Probe, ULVAC-PHI, Chigasaki, Japan) with a monochromatic Al Kα X-ray source. The thermal behavior was investigated with a PerkinElmer Pyris 1 thermobalance.

### Crystallography

Data collection for single crystals of compounds **1–3** was carried out with a Bruker D8 SMART Apex2 CCD X-ray diffractometer at room temperature, using single crystals sealed in borosilicate glass Lindeman tubes. Graphite-monochromated Mo Kα radiation (λ = 0.71073 Å) from a sealed tube was used as an X-ray source. All structures were solved by direct methods by extracting metal atom positions from the initial solution and finding other non-hydrogen atom positions in subsequent difference Fourier synthesis. All non-hydrogen atoms were refined first in an isotropic and then in an anisotropic approximation. Hydrogen atoms in structures of **1**– **3** were added by geometric calculations and refined isotropically in a riding approximation. Difficulties in location of H atoms of the OH groups and of disordered alkoxide functions in **3** resulted in B-alerts in cif-checking. The details of data collection and refinement are summarized in Table TS1. All data collections were carried out at room temperature, but the structures featured low thermal deviation parameters, which is not very unusual for Mo(VI) alkoxides.^17^

A tabletop Bruker D2 phaser diffractometer was used for powder data collection on a sample of compound **4**. The instrument’s LynxEye XE-T detector removes the fluorescence background very efficiently without significant loss of intensity. The powder samples were spread thinly upon Si zero-background disks. A beam knife was used to decrease air scattering at low angles. Data were collected in the 2θ range 10–130° with a step length of 0.016° and a total recording time of 4 h. Maximum peak intensities were about 80000 counts. Alpha-2 peaks were stripped for the refinements using the Panalytical HighScore program.

### Electrochemical Measurements

The slurry for an electrode was prepared using 70 wt % TiMoO_5_ as the active material, 20% Super P as a conducting carbon additive, and 10% PVdF as a binder with *N*-methylpyrrolidone as solvent. The slurry coated on copper foil was dried at 80 °C for 12 h under vacuum. The dried electrode was cut into circular disks of 17 mm diameter and kept at 50 °C under vacuum prior to cell fabrications.

The coin cells were fabricated inside an Ar-filled glovebox with O_2_ and H_2_O levels below 1 and 0.1 ppm, respectively. All of the electrochemical measurements were performed in a CR2032 coin cell format using Li metal as the negative counter electrode and Celgard 2500 separator. The electrolyte was composed of 1 M LiPF_6_ dissolved in an ethylene carbonate and diethylene carbonate mixture in equal ratio with 2% fluoroethylene carbonate. The galvanostatic charge–discharge measurements were performed using an Arbin BT-2000 Multichannel Battery Cycler. A Gamry Reference 600 electrochemical workstation was used for cyclic voltammetry and electrochemical impedance studies.

### *Ex Situ* XPS and XRD Measurements

The TiMoO_5_ electrode was precycled for two discharge–charge cycles prior to stopping at 0.01 V. The cycled cell was disassembled in an Ar-filled glovebox maintained with O_2_ and H_2_O levels of 1 and 0.1 ppm, respectively. The electrode was rinsed with dimethyl carbonate to remove the electrolyte and was dried before loading for XPS analysis. The XPS measurements were conducted using a Kratos AXIS Ultra DLD Imaging X-ray Photoelectron Spectrometer using a nonmonochromatic dual anode X-ray gun with Al Kα (1486.6 eV) and Mg Kα (1253.6 eV) radiation. The *ex situ* diffraction pattern was obtained using a Rigaku X-ray Powder Diffractometer with a Cu Kα X-ray source. The electrode was placed on the sample holder on a glass slide with Kapton tape over it to seal out air.

## Results and Discussion

### Synthetic Approaches to Precursor Complexes

The reactions between homometallic alkoxide species of different metals are commonly guided by Lewis acid–base interactions.^19,20^ Species of two or several high-valent early-transition-metal alkoxides usually form homometallic aggregates and do not transform into heterometallic derivatives on simple mixing. Established approaches to increase the reactivity of metal alkoxides toward complex formation are based on partially replacing the alkoxide groups with sterically easily available oxide ligands also prone to form bridging bonds between metal centers. These approaches are based on either hydrolytic—addition of controlled minor amounts of water, which was actively used in the works of Kickelbick et al.^21^—or solvolytic (solvothermal) approaches, where the mixtures are subjected to heating with (partial) decomposition of alkoxide ligands and form oxo ligands instead, actively pursued by Eslava, Wright, et al.^16,22^ From the viewpoint that especially the solvothermal approach (with or without subsequent reoxidation) was rather efficient in the synthesis of Mo-Nb^23^ and, especially, Mo-Ta^24,25^ alkoxides, we focused originally on exploiting this reaction pathway. Heating mixtures containing molybdenum alkoxide, MoO(OMe)_4_, or MoO(O^i^Pr)_4_, in a 1:1 ratio with titanium isopropoxide, Ti(O^i^Pr)_4_, with subsequent reoxidation delivered the expected bimetallic Mo(VI)–Ti(IV) derivatives **1** and **2**, but in rather low yields.
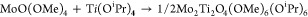
1
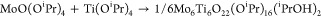
2The degree of replacement for alkoxide ligands corresponds to 1.0 per 1 equiv of two alkoxides in eq 1 and 2.67 in eq 2. This appears quite logical in the view of using only isopropoxides as reactants in eq 2, because an isopropoxide ligand is much more easily oxidized by a Mo(VI) center in comparison to a methoxide ligands. The rather low yields of crystalline products forming along with glassy or amorphous powder byproducts were indicative of the formation of a rather complex mixture of compounds. Isolated complexes **1** and **2** both had, however, the desired 1:1 ratio between metals, providing hope for finding conditions for high-yield production of the desired oxide phase precursor. Aiming at increasing the yield of compound **2**, we attempted its preparation by a hydrolytic approach, adding a stoichiometric amount of water in isopropyl alcohol to a solution of the reactants. This resulted, however, in isolation of a different product, Mo_7_Ti_7+*x*_O_31+*x*_(O^i^Pr)_8+2*x*_ (**3**), but in a quite satisfactory yield of 86%:
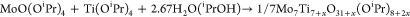
3A high yield of a bimetallic complex on microhydrolysis has in fact been observed earlier: for example, for Ba-Zr and Sr-Zr alkoxide complexes.^26^ Formation of a different product, **3** instead of **2**, was surprising but not fully unexpected, from the viewpoint of the different polarity of the medium. It is worth noting that also the degree of ligand substitution is considerably higher in **3**, equaling 3.43. This means that precipitation of the least soluble compound occurs as result of ligand redistribution. The more polar medium rich in 2-propanol can also be undergoing some additional oxidation by Mo(VI) with release of more water, which is confirmed by the emerging blue color, indicative of (hydrolyzed) Mo(V) centers.^17,18^

### Molecular and Crystal Structures of Precursors

**Figure 1:**
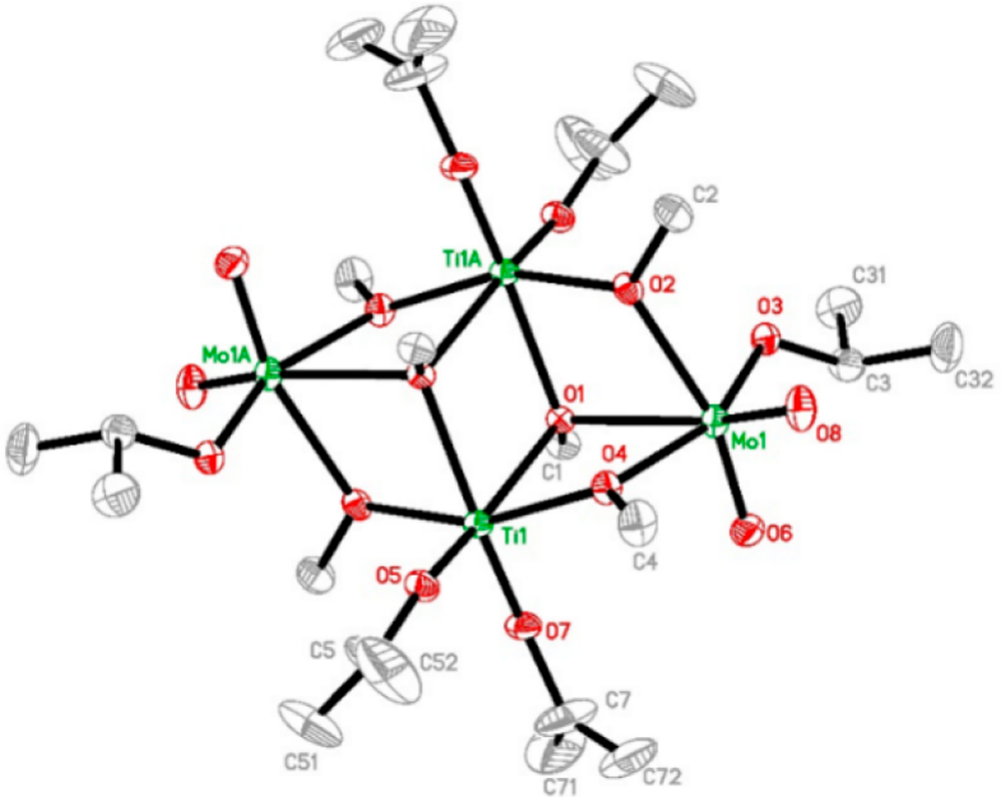
Molecular structure of the complex Ti_2_Mo_2_O_4_(OMe)_6_(O^i^Pr)_6_ (**1**). Selected bond distances (Å): Mo(1)–O(8) 1.691(5), Mo(1)–O(6) 1.694(5), Mo(1)–O(3) 1.889(4), Mo(1)–O(4) 2.115(4), Mo(1)–O(2) 2.189(4), Mo(1)–O(1) 2.249(3), Ti(1)–O(7) 1.761(4), Ti(1)–O(5) 1.764(4), Ti(1)–O(2)#1 1.955(4), Ti(1)–O(4) 1.992(4), Ti(1)–O(1)#1 2.177(3), Ti(1)–O(1) 2.195(3).

Compound **1** crystallizes in a centrosymmetric monoclinic structure built up of the centrosymmetric molecules Ti_2_Mo_2_O_4_(OMe)_6_(O^i^Pr)_6_ (**1**) and is a clathrate with two molecules of interstitial MeOH per molecule of the alkoxide (see Figure 1). It belongs to space group No. 14 in *P*2_1_/*c*, very characteristic of the layered packing of low-symmetry metal–organic molecules. The molecule of **1** is built up of two pairs of edge-sharing octahedra, Ti(O^i^Pr)_2_(μ_2_-OMe)_4_ and MoO_2_(O^i^Pr)(μ_2_-OMe)_3_, respectively. All bridging positions are occupied by smaller methoxide ligands and all terminal positions by larger isopropoxide ligands.

This fragment of flat dense packing is the most widespread structure type for heterometallic alkoxides with a 1:1 composition.^19,20^ The molecular structure results from a thermodynamically driven self-assembly, where the cation–ligand interaction is facilitated by letting the ions come closer to each other, and thus the primary ligands occupy bridging positions to minimize steric hindrance, while bulkier secondary ligands are “pressed out” to the periphery of the molecule. This type of ligand rearrangement on the formation of heterometallic complexes has recently been observed in bimetallic complexes of Zr(IV) with Ba(II) and Sr(II).^26^

The presence of bulky ligands is also responsible apparently for the appearance of **1**, as typical dioxo-molybdenum species with two equally short bonds, Mo(1)–O(8) 1.691(5) Å and Mo(1)–O(6) 1.694(5) Å, forming an O(8)–Mo(1)–O(6) angle of 104.6(3)° between them.

**Figure 2:**
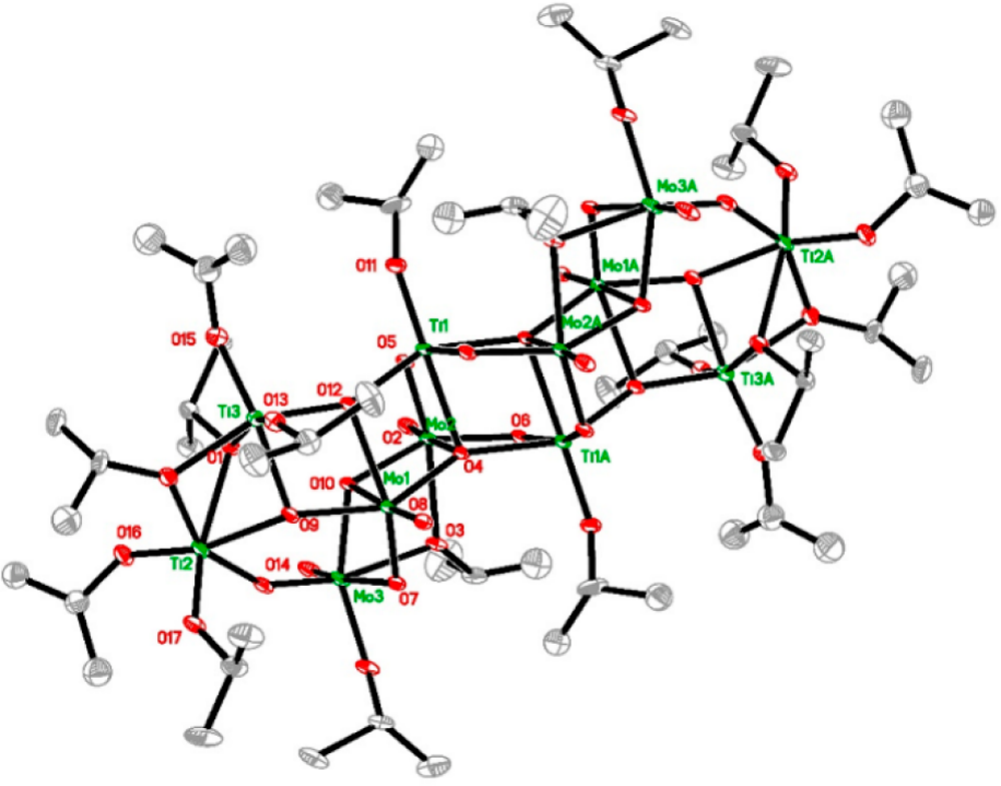
Molecular structure of the complex Ti_6_Mo_6_O_22_(O^i^Pr)_16_(^i^PrOH)_2_ (**2**). Selected bond distances (Å): Mo(1)–O(8) 1.676(6), Mo(1)–O(7) 1.729(6), Mo(1)–O(9) 1.893(6), Mo(1)–O(4) 1.943(5), Mo(1)–O(12) 2.148(5), Mo(1)–O(10) 2.484(6), Mo(1)–Ti(3) 3.156(2), Mo(1)–Ti(1) 3.2024(18), Mo(2)–O(2) 1.673(6), Mo(2)–O(6) 1.785(6), Mo(2)–O(5) 1.791(6), Mo(2)–O(10) 1.987(6), Mo(2)–O(3) 2.130(5), Mo(2)–O(4) 2.441(6), Mo(2)–Ti(1)#1 3.202(2), Mo(2)–Ti(1) 3.238(2), Mo(3)–O(14) 1.671(7), Mo(3)–O(19) 1.775(7), Mo(3)–O(18) 1.845(6), Mo(3)–O(10) 1.996(5), Mo(3)–O(3) 2.082(6), Mo(3)–O(7) 2.342(6), Ti(1)–O(11) 1.740(6), Ti(1)–O(12) 1.886(6), Ti(1)–O(5) 1.976(6), Ti(1)–O(6)#1 1.988(6), Ti(1)–O(4)#1 2.020(6), Ti(1)–O(4) 2.184(5), Ti(1)–Mo(2)#1 3.202(2), Ti(1)–Ti(1)#1 3.319(3), Ti(2)–O(17) 1.762(6), Ti(2)–O(16) 1.772(7), Ti(2)–O(20) 1.981(7), Ti(2)–O(19) 1.986(7), Ti(2)–O(9) 2.033(6), Ti(2)–O(1) 2.313(6), Ti(2)–Ti(3) 3.081(3), Ti(3)–O(13) 1.749(7), Ti(3)–O(15) 1.768(7), Ti(3)–O(12) 1.932(6), Ti(3)–O(20) 2.014(6), Ti(3)–O(9) 2.045(6), Ti(3)–O(1) 2.474(7).

The crystal structure of compound **2** is also monoclinic and centrosymmetric, belonging as well to space group No. 14, here in *P*2_1_/*n*. The molecule is a relatively large centrosymmetric construction with an ellipsoidal topology. Its asymmetric unit involves three Mo and three Ti atoms (see Figure 2), all metal atoms being octahedrally coordinated. Each Mo atom bears one doubly bonded terminal oxo ligand and is connected to the molecule mostly via bridging oxo ligands because of a considerably higher degree of alkoxide ligand substitution for oxo ligands. The coordination polyhedra for Mo atoms are Mo(1)O(μ_2_-O)(μ_3_-O)_4_, Mo(2)O(μ_2_-O)_2_(μ_3_-O)_2_(μ_2_-O^i^Pr), and Mo(3)O(μ_2_-O)_2_(μ_3_-O)(μ_2_-O^i^Pr)(O^i^Pr). The coordination arrangements for Ti atoms are Ti(1)(μ_2_-O)_2_(μ_3_-O)_3_(O^i^Pr), Ti(2)(μ_2_-O)(μ_3_-O)(μ_2_-O^i^Pr)(μ_2_-HO^i^Pr)(O^i^Pr)_2_, and Ti(3)(μ_3_-O)_2_(O^i^Pr)(μ_2_-O^i^Pr)(μ_2_-HO^i^Pr)(O^i^Pr)_2_. The position of the solvating alcohol molecule is very surprisingly bridging, Ti(2)–O(1)–Ti(3), as indicated by characteristic increased bond lengths (see Figure 2).

**Figure 3:**
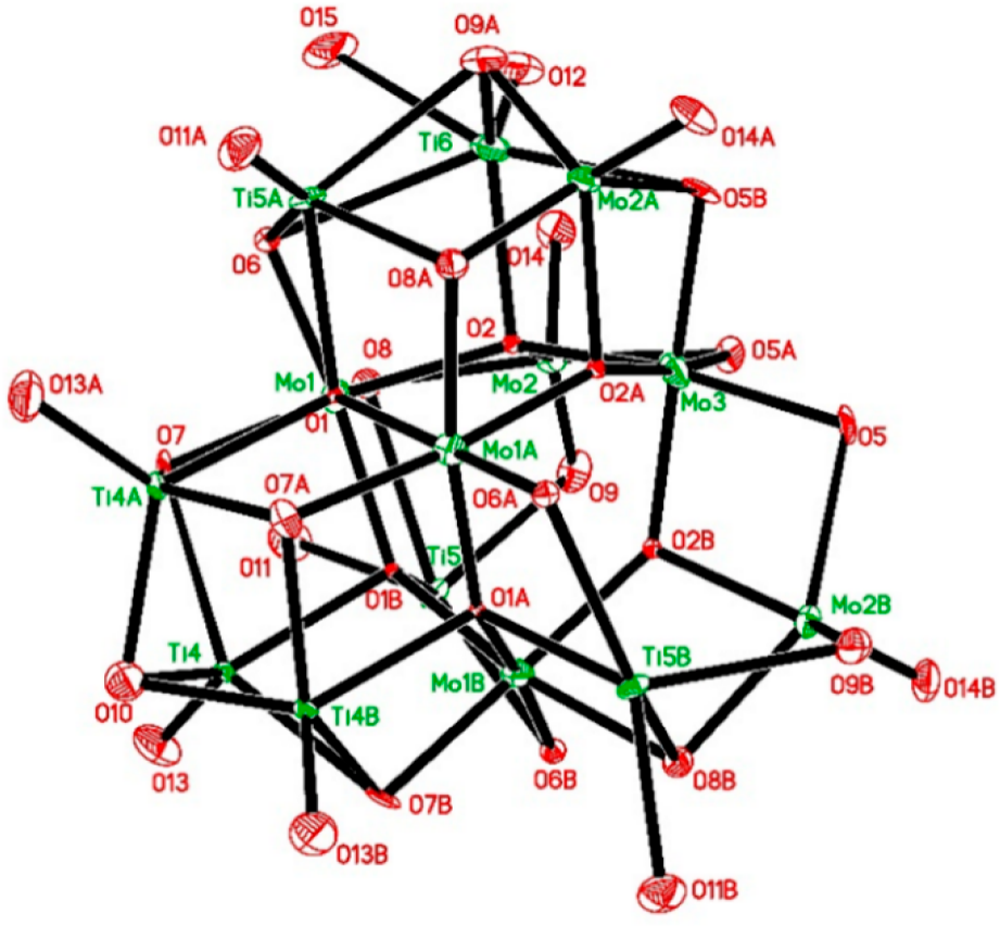
Metal–oxygen core in the structure of Mo_7_Ti_7+*x*_O_31+*x*_(O^i^Pr)_8+2*x*_ (**3**). Selected bond lengths (Å): Mo(1)–O(6) 1.855(16), Mo(1)–O(1) 1.898(6), Mo(1)–O(2) 1.952(7), Mo(1)–O(1)#1 1.975(6), Mo(1)–O(7) 1.972(9), Mo(1)–O(8) 2.040(11), Mo(1)-O(8A) 2.062(19), Mo(1)–Ti(5)#2 3.095(3), Mo(1)–Ti(4)#2 3.101(3), Mo(1)–Ti(6) 3.147(6), Mo(1)–Mo(2) 3.1689(19), Mo(1)–Ti(5) 3.205(3), Mo(2)–O(9) 1.693(13), Mo(2)–O(14) 1.694(12), Mo(2)–O(8) 2.019(12), Mo(2)–O(2) 2.033(7), Mo(2)–O(5)#2 2.134(12), Mo(2)–O(8A) 2.53(2), Mo(2)–Ti(5) 2.910(3), Mo(2)–Ti(6)#1 2.977(7), Mo(2)–Mo(3) 3.1180(14), Mo(2)–Ti(6) 3.252(7), Mo(3)–O(2)#1 1.931(7), Mo(3)–O(2)#2 1.931(7), Mo(3)–O(2) 1.931(7), Mo(3)–O(5) 1.993(10), Mo(3)–O(5)#1 1.993(10), Mo(3)–O(5)#2 1.993(10), Mo(3)–Mo(2)#1 3.1180(14), Mo(3)–Mo(2)#2 3.1180(14), Mo(3)–Ti(6) 3.149(7), Mo(3)–Ti(6)#2 3.149(7), Mo(3)–Ti(6)#1 3.149(7), Ti(4)–O(13) 1.722(15), Ti(4)–O(1)#1 2.101(6), Ti(4)–O(10) 2.086(15), Ti(4)–C(6A)#1 2.27(3), Ti(4)–O(7)#1 2.276(12), Ti(4)–O(7) 2.304(12), Ti(4)–Ti(4)#2 2.887(4), Ti(4)–Ti(4)#1 2.887(4), Ti(4)–Mo(1)#1 3.101(3), Ti(4)–Ti(5) 3.398(3), Ti(5)–O(8A) 2.039(19), Ti(5)–O(1)#1 2.125(6), Ti(5)–O(11) 2.174(16), Ti(5)–O(9) 2.328(15), Ti(5)–O(8) 2.446(12), Ti(5)–O(6)#1 2.477(17) .

Compound **3** has a noncentrosymmetric body-centered cubic crystal structure, belonging to the space group *I*4̅_3_*d*. It contains 345 Å^3^ of accessible voids (A alert in checkcif), indicating that it can incorporate up to ca. 1 ^i^PrOH molecule per molecule of the complex, which is reflected by TGA data. The observed high symmetry is very unusual for alkoxide complexes, resulting in a powerful disorder of both cations and ligands (see Figure 3).

The positions of Mo(VI) and Ti(IV) cations are not mixed with each other, but the Ti(6) position is only partially occupied, being present mostly in one of three symmetrically equal locations. The *x* value varyies between different crystals in the range ∼0.50–0.65. This indicates that the structure contains a mixture of Ti_7_Mo_7_ and Ti_8_Mo_7_ molecules in a 1:1 to 2:3 ratio. This is rare, but not unique, as in particular Ti oxo-isopropoxides when they are isolated from toluene show cocrystallization of Ti_11_ and Ti_12_ cores.^27^ This causes the composition of **3** to deviate slightly from an exact 1:1 composition with a minor excess of Ti (Mo_7_Ti_7.65_, Mo:Ti = 1:1.07). Mo(1) and Mo(3) are surrounded only by bridging oxo and alkoxo ligands and are octahedrally coordinated. Mo(2) is pentacoordinated and features essentially the dioxo fragment Mo(2)O(14)O(9), Mo(2)–O(9) 1.693(13) and Mo(2)–O(14) 1.694(12) Å, with equally long double bonds even though O(9) is involved in another much longer bond to Ti(5), Ti(5)–O(9) 2.328(15) Å. Titanium atoms are actually all pentacoordinated with one terminal alkoxide group and four bridging oxo ligands attached to each of them. The low coordination numbers result, presumably, from a high degree of alkoxide ligand substitution in this structure of 3.43, in combination with steric hindrance from the residual isopropoxy groups on the surface. In total, it is possible to conclude that the bond lengths and angles in structures **1**–**3** fall within typical intervals for Mo(VI) and Ti(IV) alkoxide complexes, respectively.^27^

The IR spectrum of **3** (see Figure S1) is consistent with the obtained structure, revealing the complex nature of Mo–O bonds with increased multiplicity by the presence of bands at 956, 934, and 914 cm^–1^. The NMR spectrum of **3** does not contradict the observed solid-state structure with 4 types of isopropoxide ligands for 8 + 2*x* groups in total in the molecule.

### Preparation and Structure of the TiMoO_5_ Phase

In order to approach the heterometallic oxide phase, a sample of single crystals of compound **3** was investigated by TGA, revealing that its decomposition occurs in the temperature interval 140–190 °C with subsequent combustion of the residual carbon at 480–500 °C (see Figure S2). These observations were used to choose the conditions for the preparation of the mixed oxide phase. The thermal decomposition of the precursor was carried out with slow heating to avoid a phase separation of the individual oxides, followed by annealing at 500 °C to achieve crystallization and remove residual carbon. The major product of this reaction was the new phase TiMoO_5_ (**4**), for which the details of structure determination are reported below. It is important to mention that the same kind of thermal treatment for an amorphous (blue) product produced by the addition of an excess of water to a 1:1 solution of molybdenum and titanium isopropoxides displayed the orthorhombic MoO_3_ as the major crystalline component (see Figure S3). This underlines the utility of using a single-source precursor in this particular case.

**Figure 4:**
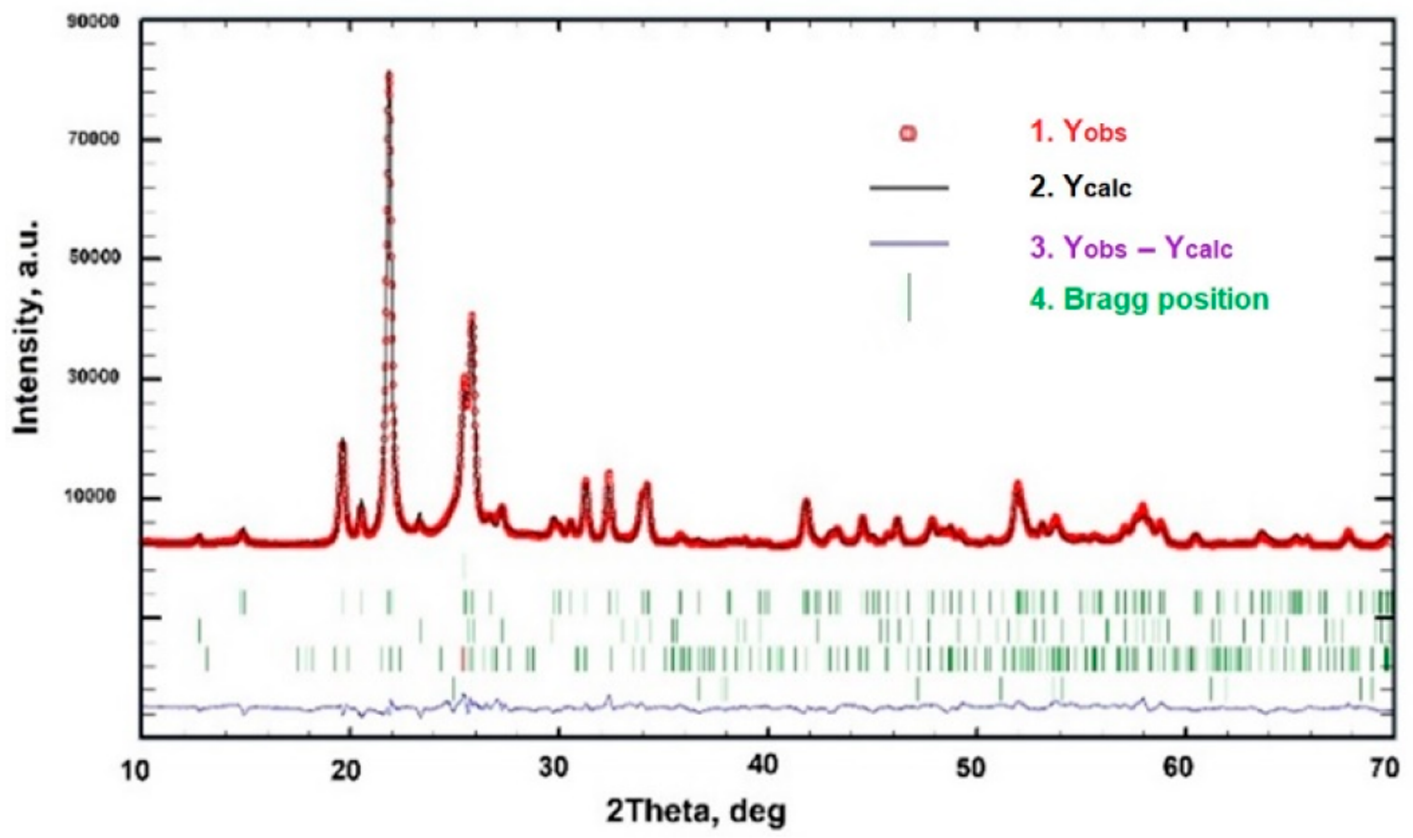
Observed, calculated, and difference X-ray powder patterns for MoTiO_5_.

**Figure 5:**
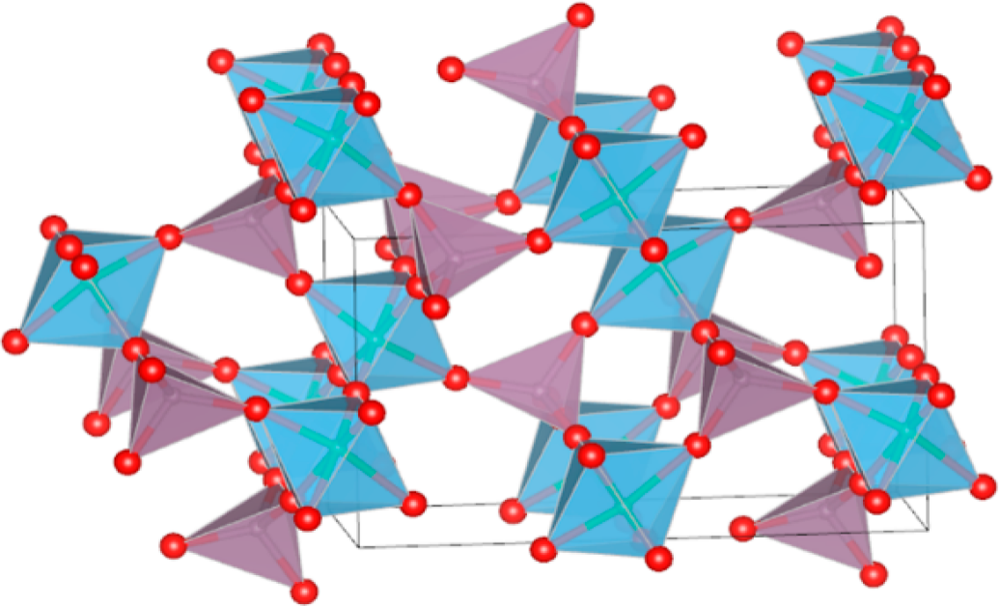
Polyhedral illustration of the MoTiO_5_ (**4**) structure. Light blue octahedra contain Ti(Mo) and violet tetrahedra Mo(Ti).

The structure of **4**, the main product of thermal decomposition of compound **3** under the reported conditions, was identified as a TaVO_5_ type phase by search matching using the PDF-4+ 2020 database.^28^ The sample was also found to contain anatase TiO_2_, Ti_6_O_11_, and MoO_3_, with refined phase fractions of 3, 20, and 4 wt %, respectively. Structural data for these were taken from the ICSD structural database.^29^ The structure of MoTiO_5_ was refined using the Rietveld method and the FullProf program (see Figures 4 and 5).^30^ The NPROF = 5 pseudo-Voigt profile shape function was used, which has 4 refinable parameters; U, V, W and η. Observed peak half-widths are given in Table S1. The peaks are broad, and the increase in the half-widths with 2θ for MoTiO_5_ indicates that it has both a substantial size and a strain of sample broadening. Berar and Lelann^31^ have suggested that the esds of refined parameters should be multiplied by a factor accounting for serial correlation in the data, this being ∼7 for the present samples. The values below are, however, not multiplied by this factor.

Data in the limited 2θ range 10–70° were used for the structure refinement, comprising 116 theoretical reflections for MoTiO_5_. A total of 37 parameters were refined, including for MoTiO_5_ 13 atomic coordinates, 1 collective temperature factor for the 2 metal sites, 1 collective temperature factor for the 4 O atoms, and 1 occupancy parameter for the metal sites that allows for a mixed occupancy of Mo and Ti on the two sites. Observed, calculated, and difference powder patterns are shown in Figure 4. The details of powder data treatment are provided in Table S2.

The χ^2^ value is 17.6. We partially attribute this large value to the good counting statistics. Residual indices for the four phases are given in Table S3, comprising for MoTiO_5_*R*_B_ = 0.067 and *R*_F_ = 0.056. The structural parameters obtained for MoTiO_5_ are given in Table 1 and derived metal–O distances in Table 2.

**Table 1:** Structural Parameters for MoTiO_5_^a^

atom	*x*	*y*	*z*	β (Å^2^)	occupancy
Ti(Mo)	0.0474(4)	1/4	0.3591(6)	2.66(7)	0.82(5) Ti/0.18(5) Mo
Mo(Ti)	0.3355(3)	1/4	0.5422(4)	2.66	0.82(5) Mo/0.18(5) Ti0
O1	0	0	1/2	0.8(2)	1.0
O2	0.1146(7)	0.0151(15)	0.162(2)	0.8	1.0
O3	0.1883(12)	1/4	0.492(2)	0.8	1.0
O4	0.4165(12)	1/4	0.3314(19)	0.8	1.0

a Space group *Pnma*, *a* = 11.8929(9) Å, *b* = 5.5231(4) Å, *c* = 6.9509(6) Å, and *V* = 456.57 Å^3^.

**Table 2:** Derived Ti(Mo)–O and Mo(Ti)–O Distances (Å) for the MoTiO_5_ Structure^a^

Ti(Mo)–O1 2×	1.784(3)	mean = 1.937
Ti(Mo)–O2 2×	2.049(12)	
Ti(Mo)–O3	1.913(15)	
Ti(Mo)–O4	2.044(15)	
Mo(Ti)–O2 2×	1.786(10)	mean = 1.777
Mo(Ti)–O3	1.785(15)	
Mo(Ti)–O4	1.754(14)	

a Shannon-Prewitt ionic radii^32^ for the relevant ions: Mo^6+^(IV) = 0.41 Å, Mo^6+^(VI) = 0.59 Å, Ti^4+^(IV) = 0.42 Å, Ti^4+^(VI) = 0.605 Å. Thus, the expected values are Ti–O6 2.01 Å and Mo–O4 1.81 Å.

**Figure 6:**
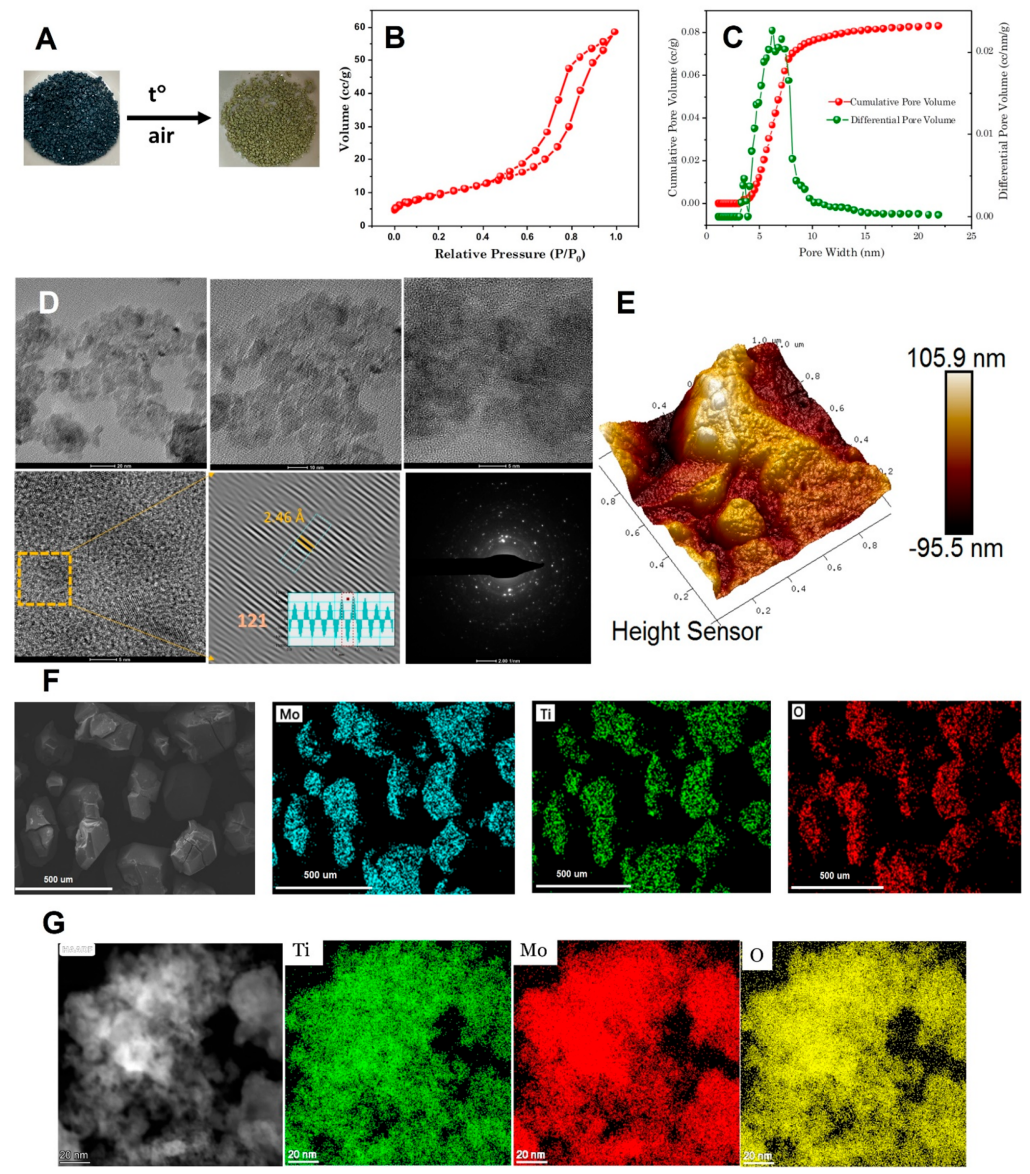
Visible transformation of crystals of **3** into meso crystals of **4** (A). Nitrogen sorption isotherm for **4** (B). Pore size distribution in the produced sample of **4** (C). HREM analysis (D). AFM image of the surface (E). SEM image and elemental mapping (F). TEM image and elemental mapping (G).

The surface area and pore distribution of TiMoO_5_ powder were evaluated using the Brunauer–Emmett–Teller (BET) method.^33^ The isotherm obtained from the N_2_ adsorption–desorption analysis is shown Figure 6B and represents a type IV isotherm, indicating the absence of micropores and the presence of a wide pore distribution along with many mesopores (2–50 nm). The material exhibited a surface area of 33 m^2^/g and a pore volume of 0.083 cm^3^/g. The pore distribution curve shown in Figure 6C indicates the absence of micropores and a pore volume from mesopores with a pore size in the range of 4–9 nm.

### Thermal Properties and Microstructure of the TiMoO_5_ Material

The titanium molybdate material **4** possesses appreciable thermal stability. According to a TGA analysis (see Figure S4), it undergoes decomposition into rutile TiO_2_,^34^ as a white powder, and orthorhombic MoO_3_^35^ (evaporating and recrystallizing separately as transparent slightly yellowish needles) phases at 730–820 °C. The synthesis of **4** occurs in a topotactic fashion: the cubooctahedral crystals of **3** change color from dark blue to light yellow (see Figure 6A) but preserve their appearance in size and shape. The transformation occurs apparently via random nucleation and crystallization. The material is built up of relatively uniform crystallites ca. 10 nm in size arranged into a mesoporous construction with a sharp pore size distribution having a maximum at 4–9 nm, as confirmed by low-temperature nitrogen sorption (Figure 6B,C), direct HRTEM (Figure 6D), and AFM (Figure 6E) observations. The surface area calculated using the BET approach was determined as 33.4 m^2^/g with a pore volume estimated using the BJH model being 0.083 cm^3^/g. The distribution of the elements remains rather uniform, as revealed by both SEM-EDS (see Figure 6F) and TEM-EDS (Figure 6G). Additional details of SEM and AFM analysis are provided in Figures FS5 and FS6.

### Electrochemical Properties of the TiMoO_5_ Material

**Figure 7:**
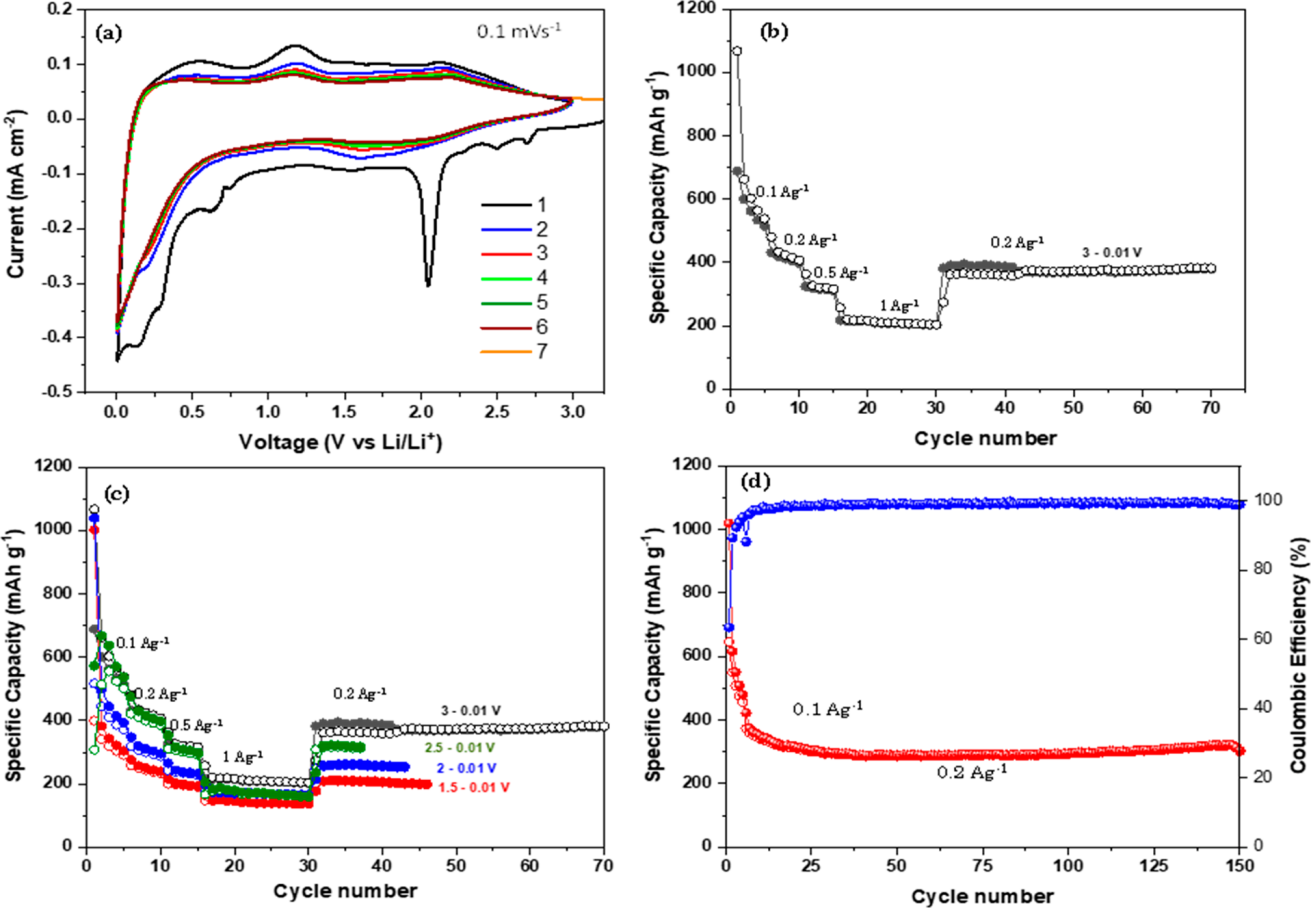
(a) Cyclic voltammogram of a Li-TiMoO_5_ cell at 0.1 mV s^–1^ (b) Rate performance in the potential window 3–0.01 V, (c) Rate performance studies for different upper cutoff potential windows. (d) Long-term cyclic stability and Coulombic efficiency for 3–0.01 V.

The electrochemical performance of TiMoO_5_ was investigated for both Li ion and Na ion anode materials. In order to understand the suitability of TiMoO_5_ for Li ions, we performed cyclic voltammetry studies in the coin cell format with Li metal as counter and reference electrodes in the potential window 3–0.01 V. Figure 7a represents the first seven cycles of cyclic voltammograms at 0.1 mV/s. The first discharge cycle shows multiple cathodic peaks at 2.1 and 0.7 V and below 0.1 V, which can be attributed to the reaction of Li with O and electrolyte decomposition to form a solid electrolyte interphase and an Li ion insertion, respectively. The absence of the sharp peaks at 2.1 and 0.7 V indicates the irreversible capacity loss due to lithium oxide and SEI formation. The overlapped cathodic and anodic peaks in the subsequent cycles indicate the reversible lithiation in TiMoO_5_, opening the possibility to use it as a conversion type anode for Li ion batteries.^36,37^ The rate performance of TiMoO_5_ for current densities varying from 100 mA g^–1^ to 2 A g^–1^ is shown in Figure 7b. As is evident from Figure 7b, the material showed a first cycle reversible capacity of 690 mAh g^–1^ and a retained discharge capacity of 200 mAh g^–1^ when the current density was increased to 1A g^–1^ when it was cycled in the potential window 3–0.01 V. The material retained 60% of its initial reversible capacity when the current density was reverted to 200 mA g^–1^. Furthermore, we studied the effect of the operating potential window on the specific capacity, rate performance, and cycle life of TiMoO_5_.

Figure 7c represents the rate performance of TiMoO_5_ obtained from constant current charge–discharge cycling on varying the upper cutoff from 1.5 to 3 V while keeping the lower potential the same at 0.01 V. The specific capacity decreased with a decrease in upper cutoff potential window, as is evident from Figure 7c. The highest value for the reversible capacity, 690 mAh g^–1^, was achieved at 100 mA g^–1^ for the potential window 3–0.01 V. However, only 400 mAh g^–1^ was obtained for 1.5–0.01 V at the same current density. This capacity difference can be attributed to the contribution from the surface reaction as well as the redox reaction above 2 V. While the material showed an increased capacity for a larger potential window, no significant difference was observed in cycle life and rate performance. The long-term cyclic stability of the material was evaluated at 200 mA g^–1^ in the potential window 3–0.01 V and is shown in Figure 7d. The cell was cycled at 100 mA g^–1^ for an initial five cycles for proper SEI formation and electrode activation. The material exhibited a reversible capacity of 648 mAh g^–1^ for the first several initial formation cycles at 100 mA g^–1^ and 380 mAh g^–1^ at 200 mAh g^–1^. The material showed an excellent cycle life of 150 cycles with 85% capacity retention.

Electrochemical impedance spectroscopy (EIS) measurements were performed to understand the electron and ion transfer resistance of the TiMoO_5_ electrode. Figure S8 depicts the Nyquist plots of TiMoO_5_ electrodes before and after cycling under delithiated conditions in the frequency range 10 mHz to 100 kHz and at an AC amplitude of 10 mV. The material exhibited series resistances of 8.8 and 5.3 Ω before and after cycling, respectively. The improved series resistance and charge transfer resistance of the cycled electrode can be attributed to the enhanced access to the electrolyte and electroactive surface area.

We further obtained cyclic voltammograms at different scan rates of 0.2, 0.5, and 1 mV s^–1^ to understand the Li ion storage mechanism. The initial seven cycles of the cyclic voltammograms at different scan rates of 0.1, 0.2, 0.5, and 1 mV s^–1^ are shown in Figure S7 in the Supporting Information. According to the power law, the peak current density is directly proportional to scan rate, *i* = *av*^*b*^, where *a* and *b* are constants.^38^ The value of *b* signifies the charge storage mechanism. A value close to 5 shows that the reaction is diffusion-controlled, indicating a bulk reaction, while *b* = 1 implies that the charge storage is through a surface-controlled reaction. Figure S9b represents the variation of peak current density with scan rate for the CV curves in Figure S9a. The values of *b* obtained for both cathodic and anodic peaks of 0.869, 0.905, and 0.828 clearly confirm a surface-controlled charge storage mechanism.

**Figure 8:**
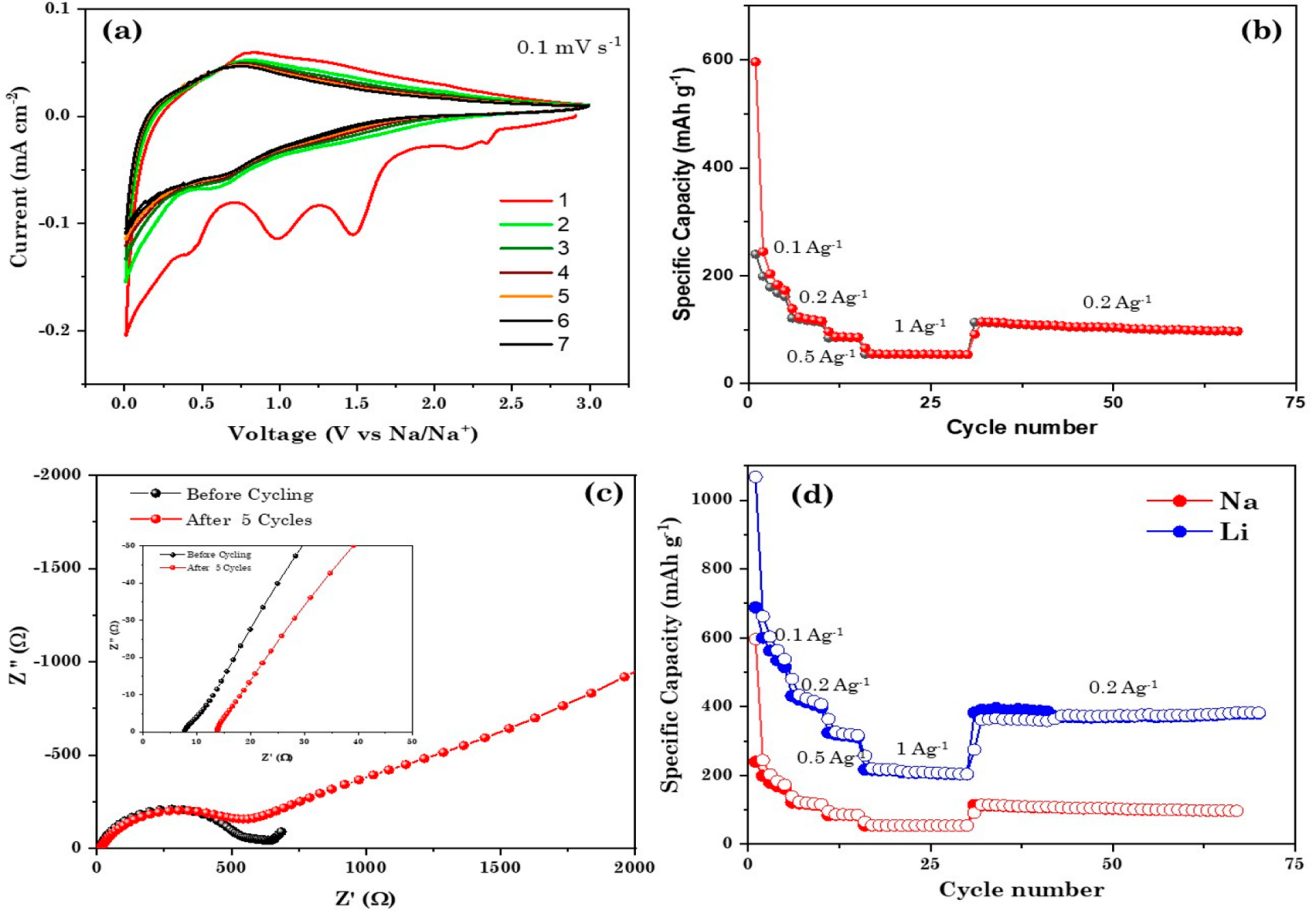
(a) Initial seven cycles of cyclic voltammogram at 0.1 mV s^–1^. (b) Rate performance at current densities ranging from 0.1 to 1 A g^–1^. (c) Nyquist plot obtained from the EIS studies in the frequency range 10 mHz to 100 kHz. (d) Comparison of the rate performance of TiMoO_5_ as Li ion and Na ion anodes for current densities from 0.1 to 1 A g^–1^.

Furthermore, we have studied TiMoO_5_ as an anode for Na ion batteries. The electrochemical measurements were performed in the coin cell format using Na metal as the negative electrode and a Whatman glass fiber separator. NaClO_4_ (1 M) dissolved in an EC/DEC mixture in a 1:1 ratio with 2% FEC additive was used as the electrolyte. Figure 8a shows the initial seven cycles of cyclic voltammograms of Na-TiMoO_5_ at 0.1 mV s^–1^ in the potential window 3–0.01 V. The first discharge (sodiation) cycle exhibits multiple peaks at around 1.5, 0.9, and 0.4 V. The absence of these peaks in the subsequent cycles indicates stable SEI formation and the absence of parasitic reactions. The broad peak in the range 0.8–1 V during the first cycle can be attributed to the electrolyte decomposition to form an SEI layer. The absence of any prominent peaks from the second cycle onward indicates surface-controlled/pseudocapacitive Na ion storage in TiMoO_5_.^39^ The rate capabilities of TiMoO_5_ at higher current densities are shown in Figure 8b. The material delivered the first reversible capacity of 240 mAh g^–1^ at 100 mA g^–1^ and retained only 54 mAh g^–1^ when the current density was increased to 2 A g^–1^. Moreover, a capacity of 110 mAh g^–1^ was maintained after 30 cycles when the current density was kept at 200 mA g^–1^. The material exhibited a fast capacity fade in spite of the high reversible capacity. Figure 8c depicts the Nyquist plots of pristine TiMoO_5_ and TiMoO_5_ after five continuous charge–discharge cycles under desodiated conditions in the frequency range 10 mHz to 100 kHz and an AC amplitude of 10 mV.

The material exhibited series resistances of 8.5 and 14.1 Ω before and after cycling, respectively. An increase in the impedance was observed in the case of cycled electrodes. This can be associated with the fast capacity fade and low rate performance. Figure 8d compares the electrochemical performances of TiMoO_5_ as an anode for Li ion and Na ion batteries. While the material showed promise for Li ion batteries with high reversible capacity and rate performance, a significantly lower capacity was obtained for the Na ion battery.

### Transformation of TiMoO_5_ Material on Lithiation

The change in the oxidation state during the charge–discharge process was investigated using an *ex situ* XPS analysis. Figure S10 depicts the XPS spectra of pristine and lithiated TiMoO_5_ for the elements Ti and Mo. The deconvoluted Ti 2p spectrum shown in Figure S10a exhibits two peaks centered at 459 and 464.7 eV corresponding to Ti 2p_3/2_ and Ti 2p_1/2,_ respectively, in the Ti^4+^ oxidation state.^38^ Likewise, the two peaks positioned at 232.9 and 236 eV in Figure S10b correspond to Mo 3d in the Mo^6+^ oxidation state.^40^ The Ti 2p XPS data for the lithiated TiMoO_5_ shown in Figure S10c present two peaks centered around 461.3 and 465.4 eV corresponding to Ti 2p of Ti in a 4+ oxidation state. This indicates the absence of any change in the oxidation state of Ti during the charge–discharge process. The proposed hypothesis about the possibility of reducing the material while maintaining its morphology via an unaltered TiO_2_-derived component thus appears valid.

The structural transformation was also followed by X-ray diffraction on the sample precycled for two discharge–charge cycles prior to stopping at 0.01 V. The cycled cell was disassembled in an Ar-filled glovebox maintained at O_2_ and H_2_O levels of 1 and 0.1 ppm, respectively. The electrode was rinsed with dimethyl carbonate to remove the electrolyte and was dried inside the glovebox. The diffraction pattern was obtained using a Rigaku X-ray Powder Diffractometer with a Cu Kα X-ray source. The electrode was placed on the sample holder on a glass slide with Kapton tape over it to seal out air. Figure S11 shows the *ex situ* XRD of the lithiated TiMoO_5_ electrode and pristine TiMoO_5_ powder. The altered peak positions in the lithiated TiMoO_5_ indicate a phase change after lithiation. Additionally, the significant peak intensity reduction, change in the peak position, and significant broadening imply a reduction in the domain size due to the expansion/compression of the material originating from a volume change during the lithiation and delithiation. This is consistent with the behavior of other conversion type anodes known in the literature.

## Conclusions

A single-source alkoxide precursor approach was proved to be efficient in producing a new mixed oxide nanomaterial that appeared inaccessible via a traditional solid-state synthesis, and even (in this work) a sol–gel synthesis. The isolation of the individual precursor and its controlled thermal decomposition were indispensable for production of the desired oxide phase in nanosize. The produced material possesses excellent thermal stability and demonstrates several reduction steps on lithiation, associated with the reduction of Mo(VI) cations, but not Ti(IV), allowing its use as a conversion type anode for Li ion batteries with considerable morphological stability on cycling. The charge storage has, however, a surface capacitive character for Na, making the material less attractive for use in Na ion batteries.

## Supporting Information Available

IR spectrum of compound **3**, TGA for compound **3** in air, XRD for the thermal treatment product of sol–gel synthesis, using freshly produced solution analogous to that producing compound **3**, TGA for compound **4** in air, SEM and AFM images for compound **4**, summary of data collection and refinement details for compounds **1**–**3**, Rietveld refinement results for contributing phases in a sample of **4**, and additional details of electrochemical characterization of the function of **4** as anode in Li ion batteries (PDF)

## Accession Codes

CCDC 2035031–2035033 contain the supplementary crystallographic data for this paper. These data can be obtained free of charge via www.ccdc.cam.ac.uk/data_request/cif, or by emailing data_request@ccdc.cam.ac.uk, or by contacting The Cambridge Crystallographic Data Centre, 12 Union Road, Cambridge CB2 1EZ, UK; fax: +44 1223 336033.

## Abbreviations

SEM: scanning electron microscopy
TEM: transmission electron microscopy
EDS: energy dispersion spectroscopy
IR: infrared spectroscopy
NMR: nuclear magnetic resonance spectroscopy
