# Supplementary material for: Single-Source Alkoxide Precursor Approach to Titanium Molybdate, TiMoO5, and Its Structure, Electrochemical Properties, and Potential as an Anode Material for Alkali Metal Ion Batteries
Source: Inorg Chem. 2021 Feb 22;60(6):3593–603. doi: 10.1021/acs.inorgchem.0c03087 (PMC8023665; doi:10.1021/acs.inorgchem.0c03087)
Supplement: Supplementary file 1 — ic0c03087_si_001.pdf [file ic0c03087_si_001.pdf]

Supplementary materials

Single-source alkoxide precursor approach to titanium molybdate, $TiMoO_5$, its structure, electrochemical properties and potential as anode material for alkali metal ion batteries

Hiroaki Uchiyama,† Ŧ Dhanya Puthusseri,‡ Jekabs Grins, Daniel Gribble,‡ ╠ Gulaim A. Seisenbaeva,†* Vilas G. Pol,‡* Vadim G. Kessler†*

† Department of Molecular Sciences, BioCenter, Swedish University of Agricultural Sciences, Box 7015, SE-750 07 Uppsala, Sweden; Ŧ Kansai University, 3-3-35 Yamate-cho, Suita-shi, Osaka, JP 564-8680; ‡ Materials Science and Engineering, Purdue University, West Lafayette, IN 47907, USA; ╠ Department of Materials and Environmental Chemistry, Stockholm University, SE-106 91 Stockholm, Sweden.

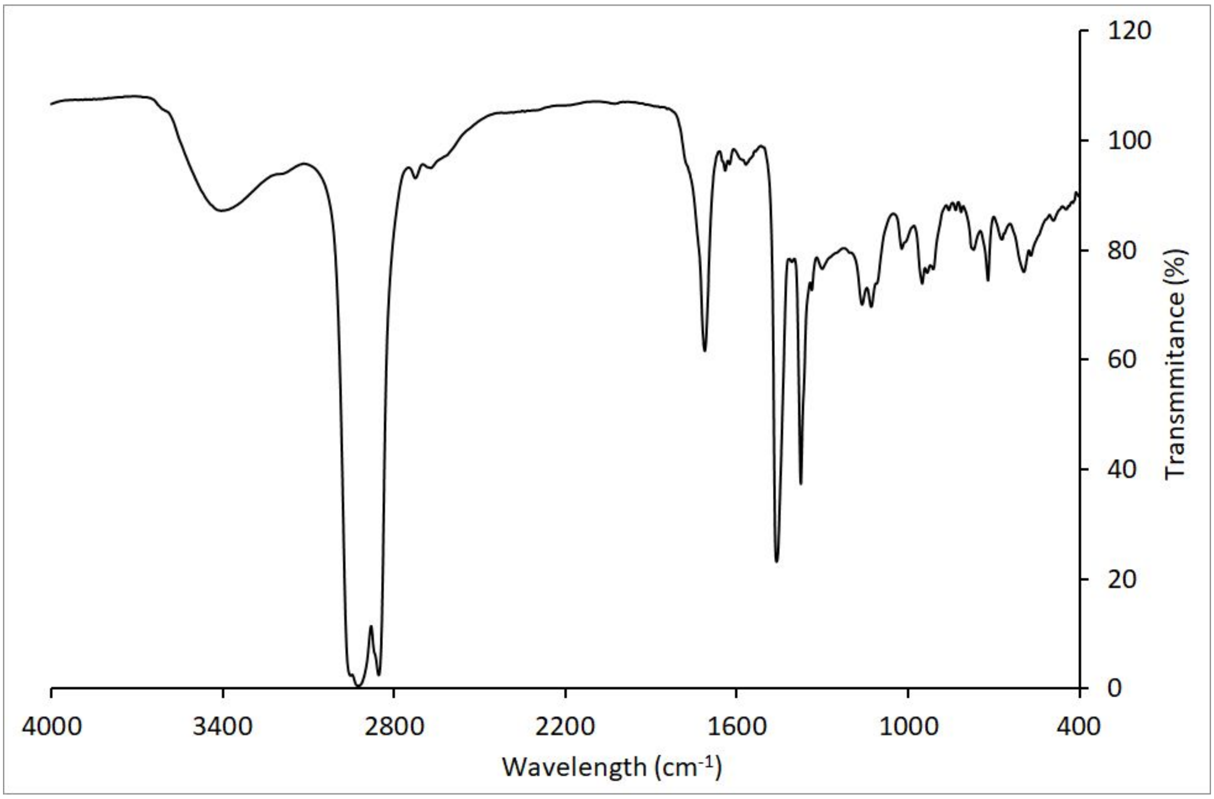

Figure S1. IR spectrum of the compound **3**.

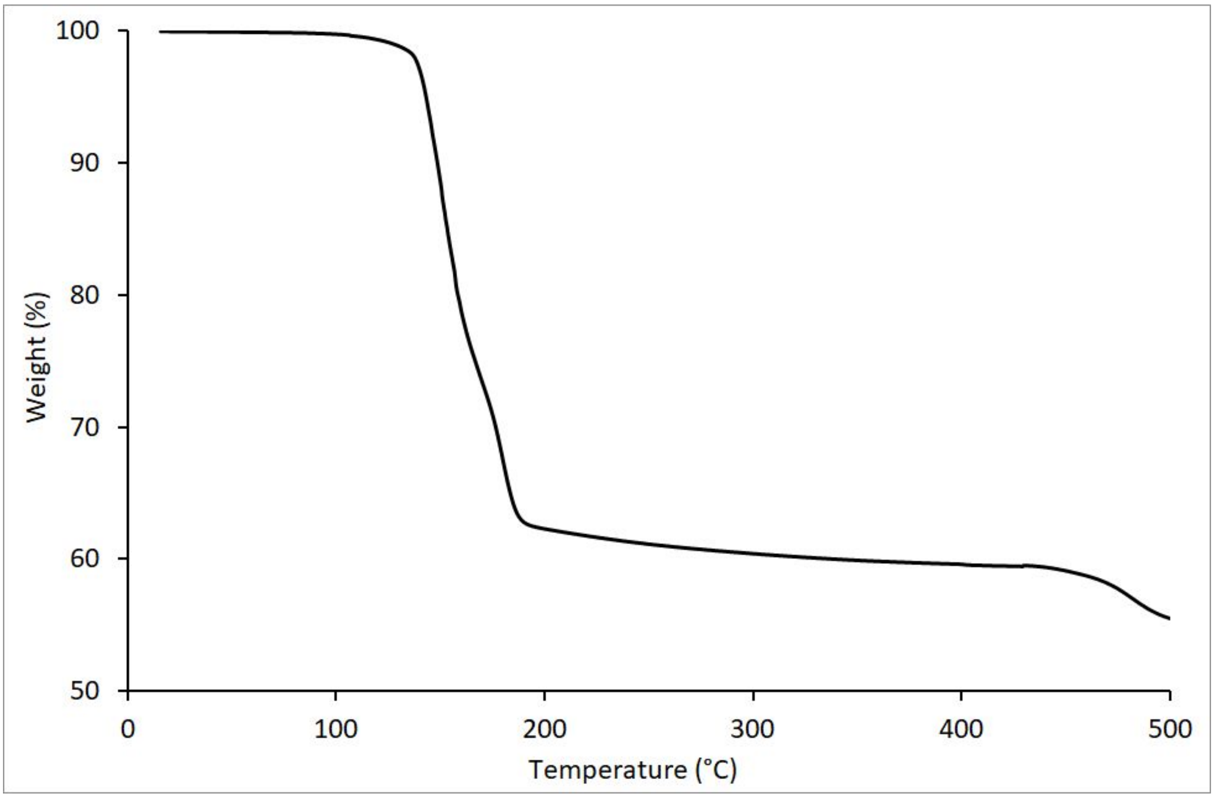

Figure S2. TGA for compound **3** in air.

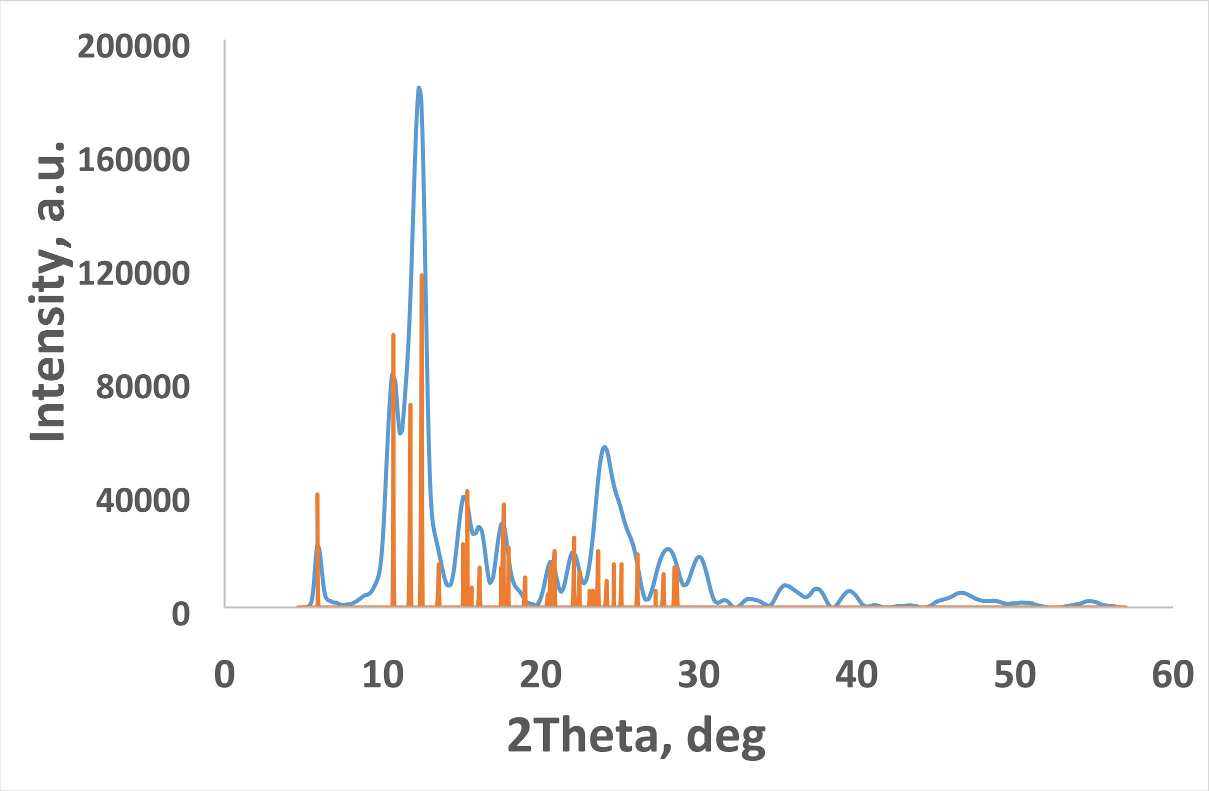

Figure S3. XRD pattern of the product of sol-gel synthesis starting from parent solution to 3 matched by the pattern of α-MoO_3, PC-PDF-00-505-0508.

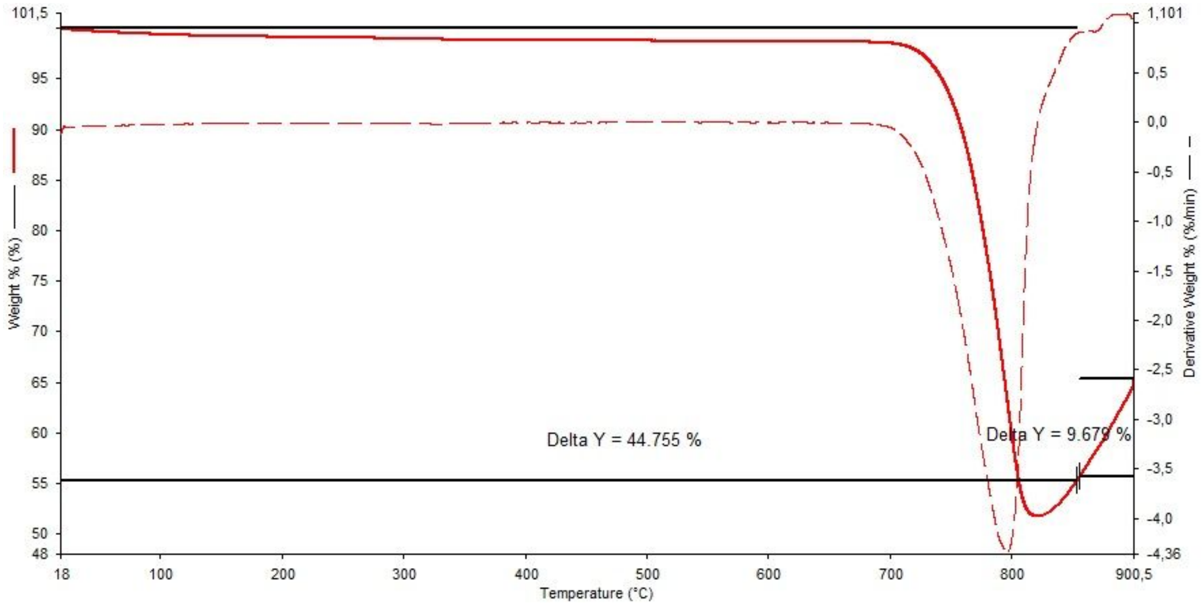

Figure S4. TGA of compound 4 in air (the re-increase in mass stays for re-precipitation of sublimed MoO_3 on the wire of the thermobalance).

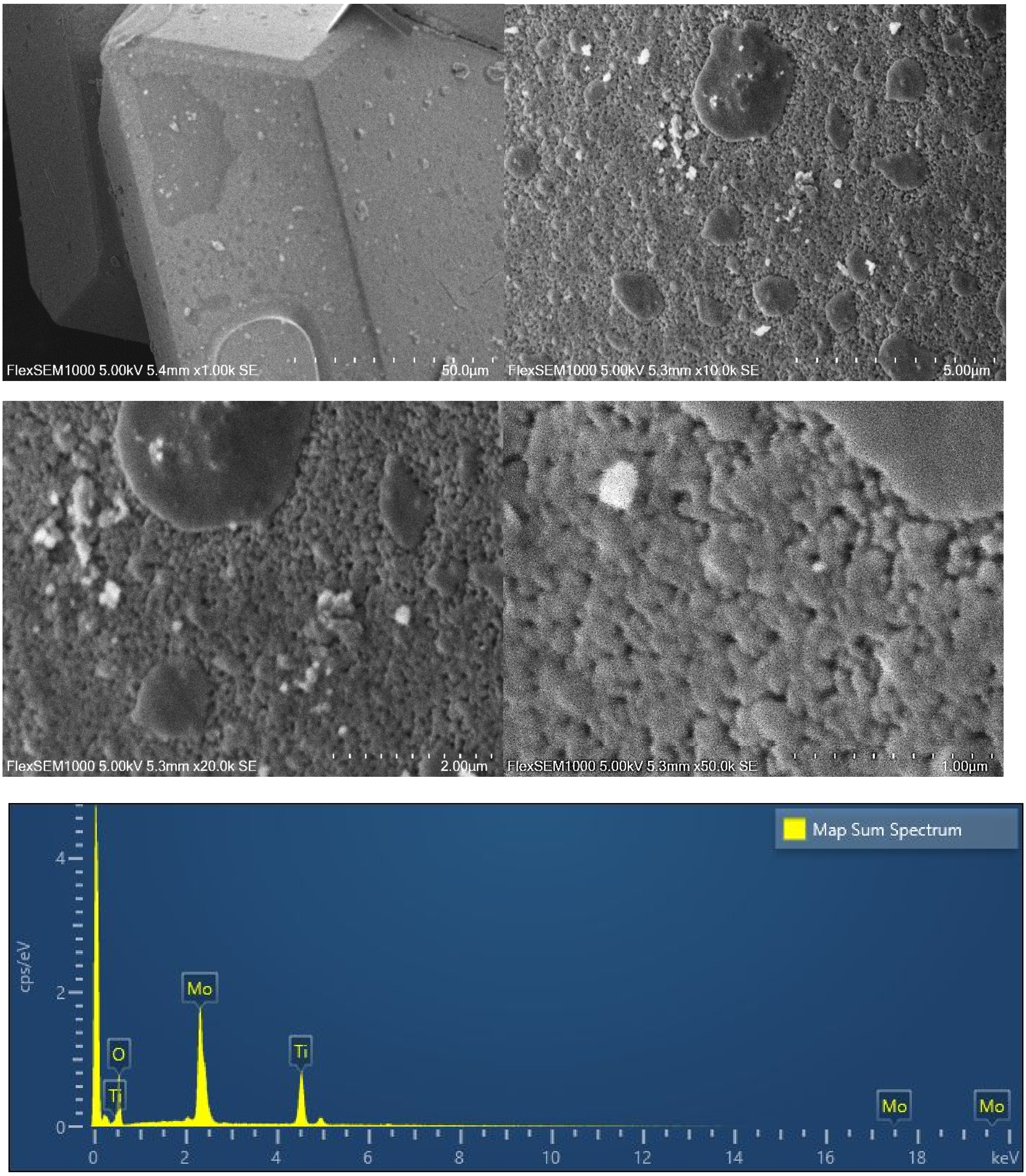

Map Sum Spectrum				
**Element**	**Line Type**	**Weight %**	**Weight % Sigma**	**Atomic %**
Ti	K series	21.50	0.36	13.60
O	K series	39.06	0.64	73.95
Mo	L series	39.44	0.55	12.45
Total		100.00		100.00

Figure S5. SEM-EDS analysis of compound **4**.

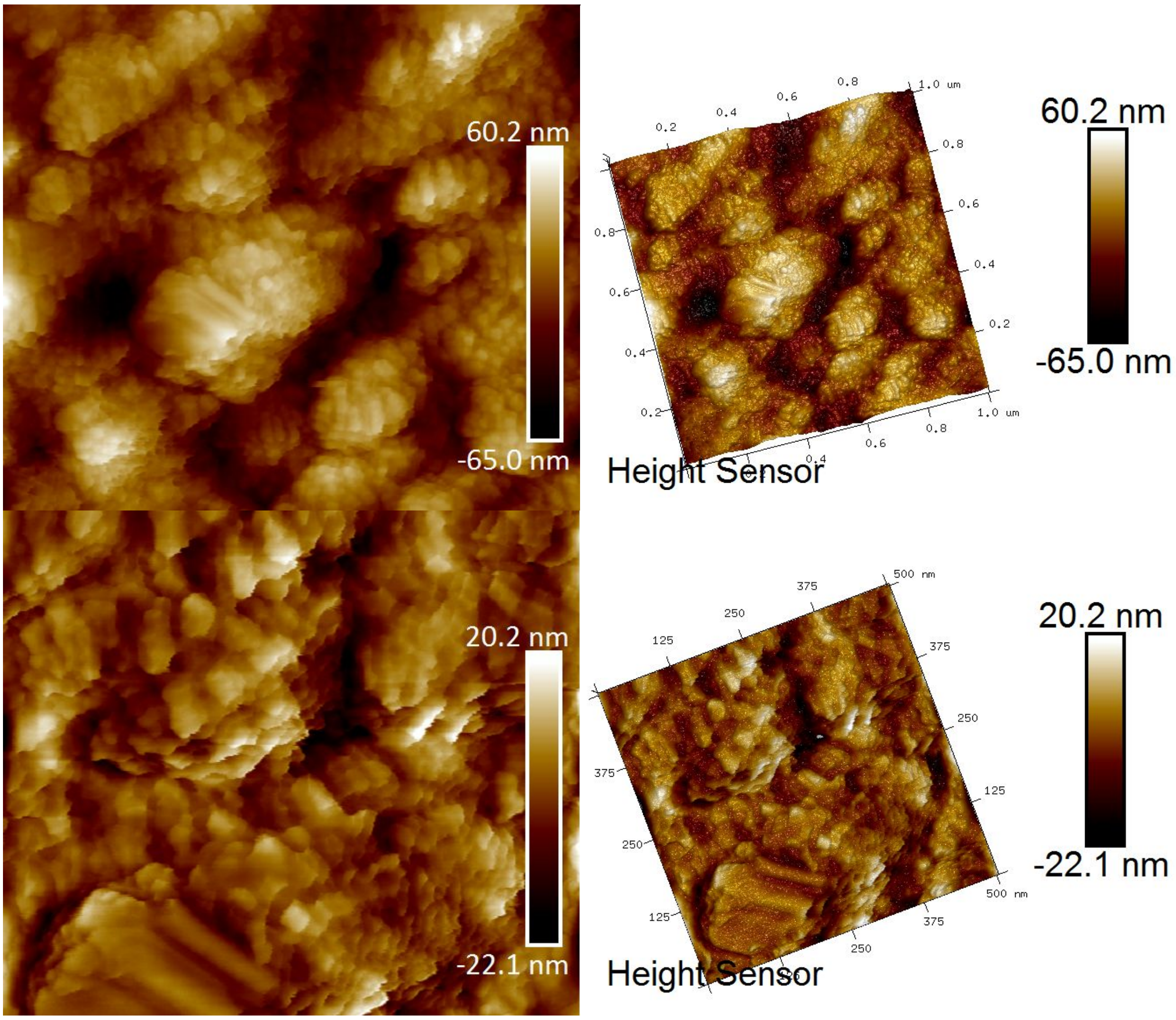

Figure S6. AFM images of the sample of **4**.

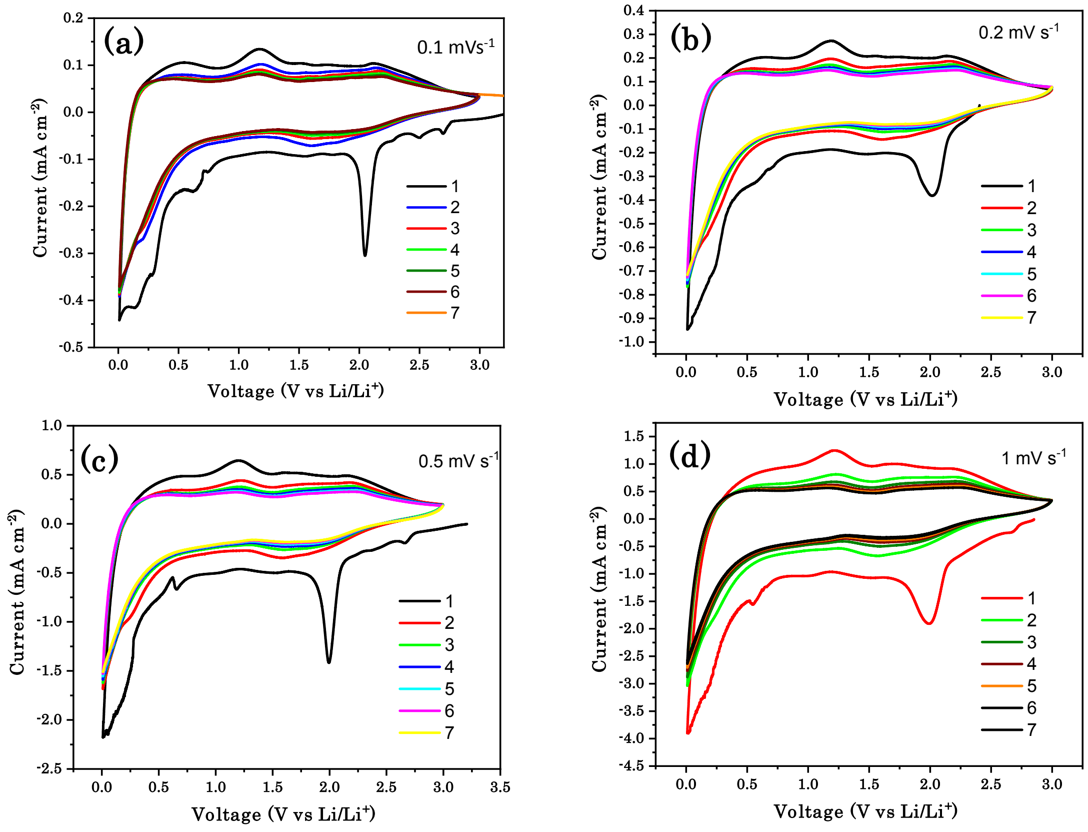

Figure S7: First seven cycles of the cyclic voltammogram of Li-$TiMoO_5$ cell at different scan rates 0.1, 0.2, 0.5 and 1 mVs^{-1}.

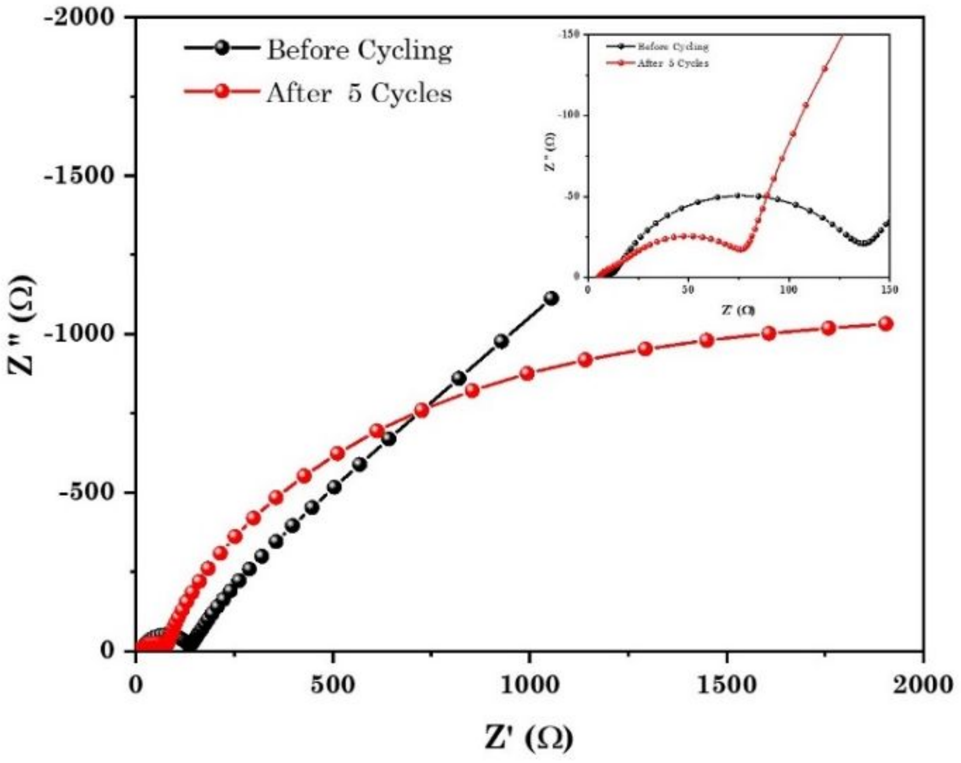

Figure S8. Nyquist plot obtained from the EIS measurement in the frequency range 10 mHz – 100 kHz. Inset is the magnified version in the high frequency region.

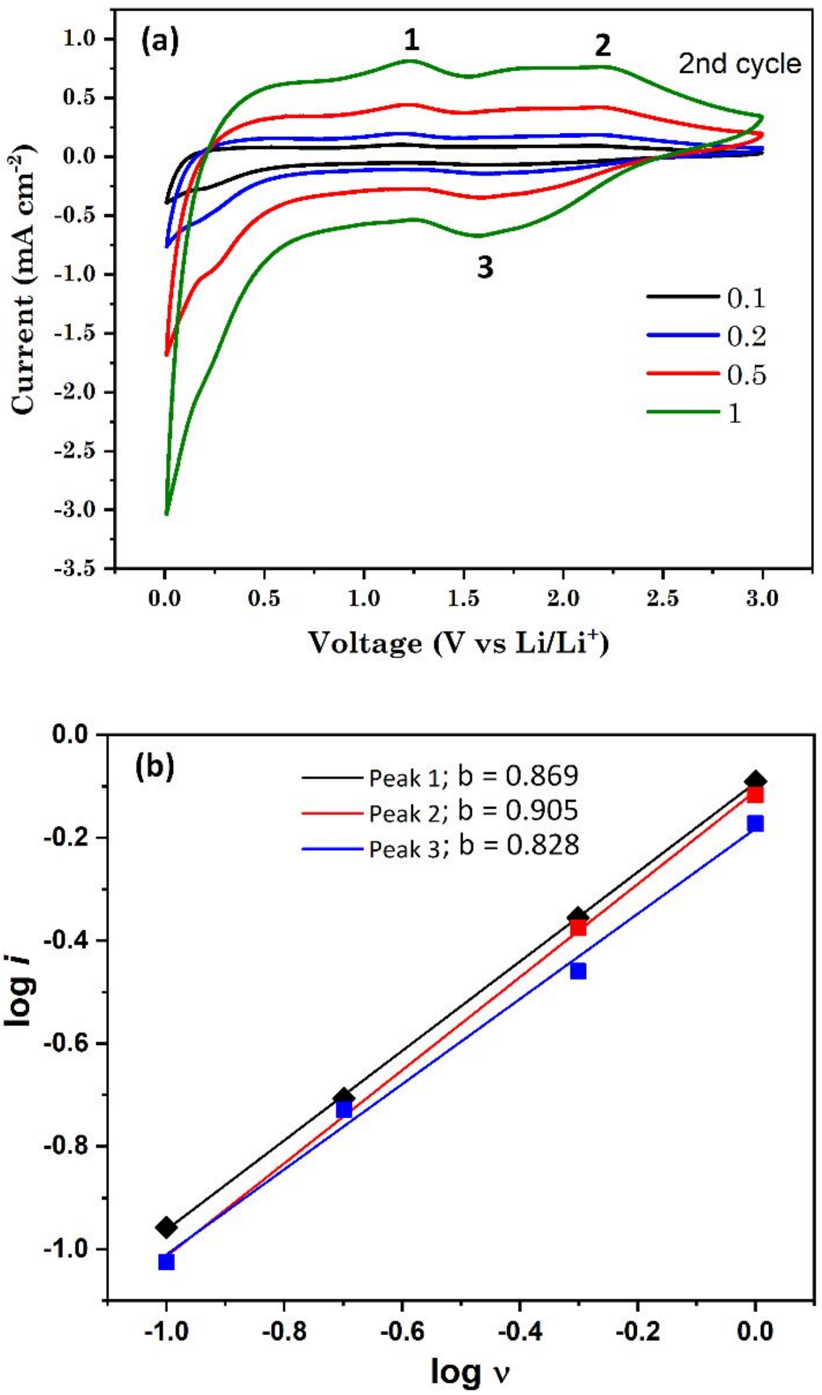

Figure S9. (a) second cycle of cyclic voltammogram at different scan rate 0.1 – 1 mVs^{-1}, (b) relationship between the peak current density and scan rate in logarithmic scale for different peaks labelled in CV.

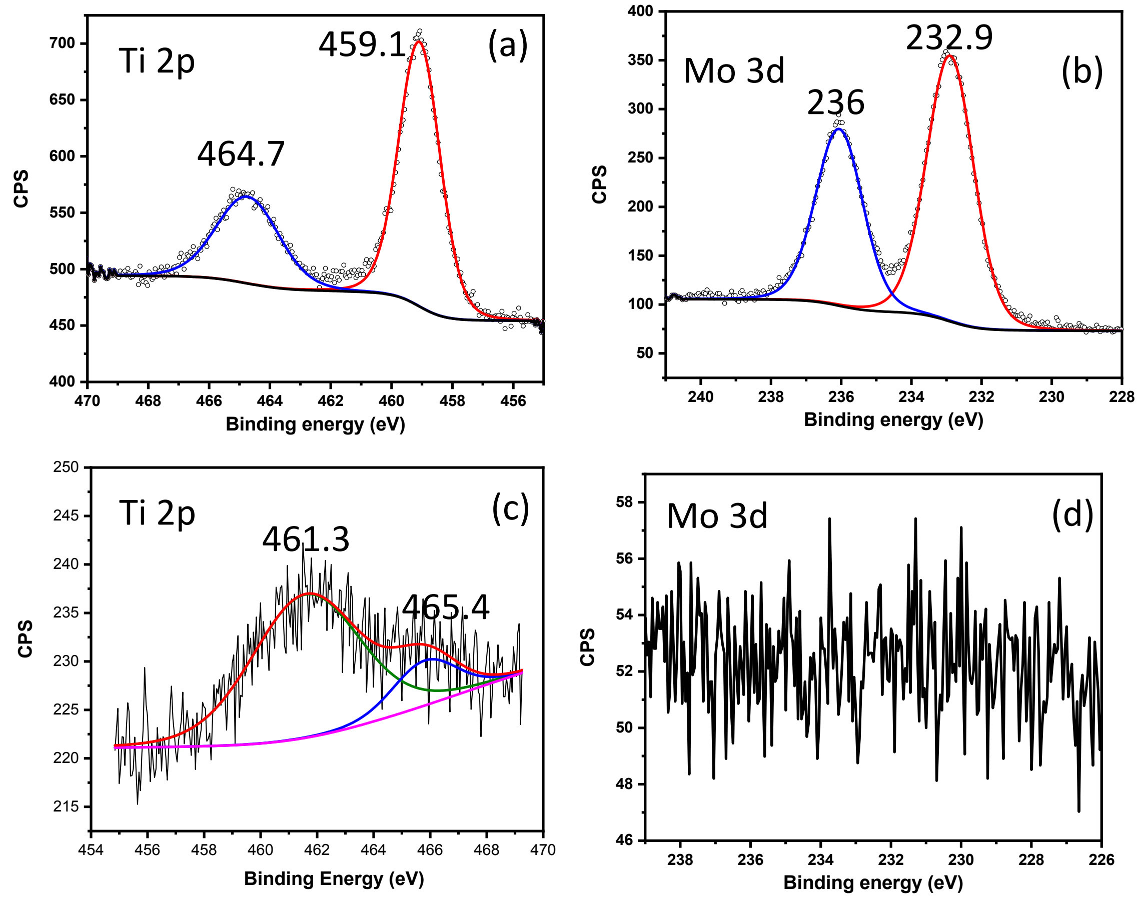

Figure S10. X-ray photoelectron spectra for (a and b) pristine $TiMoO_5$ and (c and d) lithiated $TiMoO_5$.

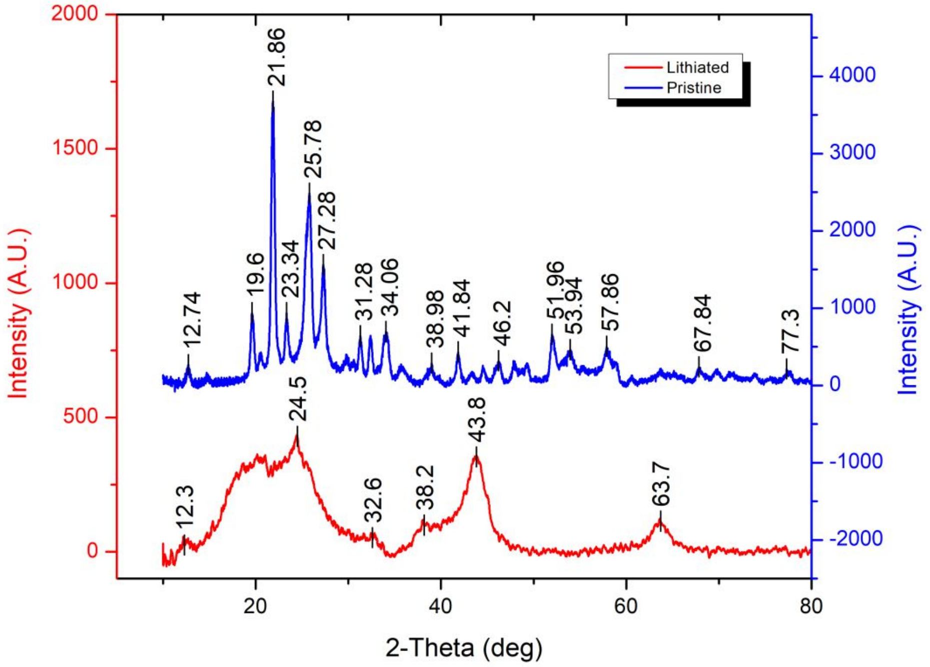

Figure S11. Powder X -ray diffraction pattern of pristine $TiMoO_5$ (blue) and $TiMoO_5$ after discharged to 0.01V vs Li (red).

TABLE S1. Details of data collection and refinement for compounds 1-3.

Chemical composition	$C_{26}H_{68}$-$Mo_2O_{18}Ti_2$(**1**)	$C_{54}H_{128}$-$Mo_6O_{40}Ti_6$(**2**)	$C_{24.50}H_{56}$-$Mo_7O_{38.50}Ti_{7.50}$(**3**)
Formula weight	956.48	2280.60	1997.52
Crystal system	Monoclinic	Monoclinic	Cubic
Space group	P2(1)/c	P2(1)/n	I-43d
μ/mm-1	0.940	1.426	2.025
R1	0.0403	0.0636	0.0838
wR2	0.1224	0.1562	0.2387
a/Å	11.121(3)	9.730(5)	29.704(4)
b/Å	17.533(5)	24.791(12)	29.704(4)
c/Å	12.084(3=	18,313(9)	29.704(4)
α/°	90	90	90
β/°	105.968(3)	98.254(6)	90
γ/°	90	90	90
V/Å3	2265.1(10)	4372(4)	26209(6)
T/K	296(2)	296(2)	296(2)
Z	2	2	16
No. of independent reflections	4096	7685	3647
No of observed reflections	3421 [I>2sigma(I)]	4937 [I>2sigma(I)]	3458 [I>2sigma(I)]

Table S2. Observed peak half-widths Hw (°).

Phase	Hw at 20°	Hw at 70°
$MoTiO_5$	0.26*	0.52*
TiO_2	0.26	0.52
Ti_6O_{11}	0.42*	0.62*
MoO_3	0.26*	0.52*

* Restrained to be the same

Table S3. Residual indices for phases, %.

Phase	R_B	R_F
$MoTiO_5$	6.7	5.6
TiO_2	4.0	2.8
Ti_6O_{11}	14.1	7.6
MoO_3	13.1	9.1
